# Low‐Temperature Single‐Step Inkjet‐Printed Metallic Patterns With Self‐Regulated Vertical Compositional Gradient

**DOI:** 10.1002/smtd.202401371

**Published:** 2025-05-15

**Authors:** Ye Zhou, Petra Vasko, Yujiang Zhu, Jingyan Wang, Curran Kalha, Anna Regoutz, Adham Hashibon, Yanlong Tai, Gi Byoung Hwang, Caroline E. Knapp

**Affiliations:** ^1^ Department of Chemistry University College London 20 Gordon Street London WC1H 0AJ UK; ^2^ Department of Chemistry University of Helsinki A. I. Virtasen aukio 1, P.O. Box 55 Helsinki 00014 Finland; ^3^ Institute of Material Discovery UCL East Marshgate 7 Sidings Street London E20 2AE UK; ^4^ Shenzhen Institutes of Advanced Technology Chinese Academy of Sciences Shenzhen 518055 China; ^5^ Department of Chemistry Inorganic Chemistry Laboratory Oxford OX1 3QR UK

**Keywords:** DFT, glucose sensing hybrid inks, inkjet printing, self‐regulated structures, structure optimization

## Abstract

For the rapidly growing demands and expanding range of applications of printed electronics in medicine lower processing temperatures and simpler steps are preferred to minimize the fabrication processes onto a range of substrates. Various hybrid inks are formulated for fabricating multi‐compositional functional patterns with fewer manufacturing processes. However, most hybrid inks can only form patterns with fully‐mixed compositional distribution. This study proposes a novel hybrid metal‐based ink formulation pathway and develops a particle‐free Ag‐Cu hybrid metal‐organic decomposition (MOD) ink. When sintering under N_2_ the in situ formed Ag and Cu nano‐particulates during the sintering process self‐regulate into a unique vertical compositional gradient with Cu dominant on top and the majority of Ag existing beneath. Highly conductive (1.88 ± 0.7 × 10^6^ S m^−1^) metallic patterns are fabricated by single‐step inkjet printing at low temperature (<150 °C) on both rigid and cellulose fiber substrates. When sintered under air a porous CuO layer is generated on the surface with high electrocatalytic activity with glucose (stable for over 2 h of continuous measurement). This work shows the feasibility of fabricating a glucose sensor including electrode layer and functional layer by single‐step printing.

## Graphical abstract

This study combines analysis of DFT modelling of complex structure decomposition studies with hybrid ink formulation resulting in inkjet printing of a range of useful electronic materials. The formulated hybrid MOD ink is exploited to form self‐regulated layered pattern structures with vertical compositional gradient at low temperature.

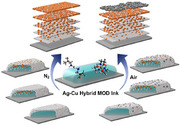

## 1 Introduction

Inkjet printing of metal‐based inks is considered to be promising for producing precise highly conductive features on flexible electronics^[^ ^1^ ^]^ and minimizing manufacturing steps cost and electrical waste (E‐waste) generation.^[^ ^2^, ^3^ ^]^ Due to the significant growing demands of light‐weight low‐cost,^[^ ^4^ ^]^ and flexible wearable devices,^[^ ^5^, ^6^ ^]^ inkjet printable metal‐based inks have started to be applied in wearable thin‐film functional printed electronics,^[^ ^7^, ^8^, ^9^ ^]^ such as electrical skins (E‐skin) thin film electrodes^[^ ^10^ ^]^ within wearable thin‐film sensors,^[^ ^11^, ^12^ ^]^ wearable capacitors^[^ ^13^ ^]^ or even smart contact lenses.^[^ ^14^, ^15^ ^]^ For these diverse applications with various types of substrates,^[^ ^16^ ^]^ the printable metal‐based inks may be required to have lower sintering temperatures to be deposited on thermally sensitive substrates,^[^ ^17^, ^18^ ^]^ or could directly fabricate functional patterns with complex vertical micro/nano structures to simplify fabrication steps^[^ ^19^ ^]^ to increase the producing efficiency and reduce the impact of manufacturing process to substrates.

In recent years metal‐organic decomposition (MOD) ink is becoming popular both in industry and research as a novel alternative to their nanoparticle‐based equivalents.^[^ ^20^, ^21^ ^]^ The particle‐free MOD inks can be smoothly and accurately printed through nozzles without a clogging issue. In addition colloidal stabilizing agents or additives are not necessary.^[^ ^18^ ^]^ This indicates that these inks can be sintered at lower temperatures (<150 (C) making them ideal for printing onto paper cellulose fiber and synthetic skins.^[^ ^22^ ^]^ Traditional metal‐based inks are typically comprised of one type of dispersed or dissolved conductive metal‐based components in a liquid solvent.^[^ ^23^, ^24^ ^]^ Multiple steps of printing with different inks are required to fabricate a functional electronic device. Hybrid metal‐based inks which contain two or more metal‐based components were developed to break through the limitations of particular components properties, and as such were used to fabricate micro/nano multi‐compositional structures within fewer steps. Gamerith et al. (2007) used both NP‐ and MOD‐based inks to synthesize a hybrid ink by dispersing Ag and Cu NPs in Ag MOD precursor solution.^[^ ^25^ ^]^ The mixed Ag/Cu NPs produced a high electrical conductivity of printed patterns and the MOD precursor structure provided interconnections with polymers and made the ink compatible with all‐solution‐based OFET processing. In addition, several Ag‐Cu core‐shell hybrid NP inks were fabricated by dispersing Cu NPs into Ag salt solutions.^[^ ^26^, ^27^, ^28^ ^]^ The trans‐metalation reaction between Cu atoms and Ag ions formed silver‐protecting layers on Cu NP surfaces to overcome the oxidation problem of Cu NP‐based inks.

During the sintering process of a MOD ink elemental metallic particulates are reduced from metal ions and moved along the internal flows in the liquid phase before deposition on the substrate.^[^ ^24^ ^]^ Theoretically a MOD ink's particulate generation process and printed pattern structures could be flexibly controlled by adjusting ink formulations and sintering conditions.^[^ ^29^, ^30^ ^]^ For a well‐designed MOD hybrid ink complex micro/nanostructures for certain functions could be directly controlled and sintered into patterns through inkjet printing technology.^[^ ^31^ ^]^ Li et al. (2022) synthesised particle‐free self‐organizing Cu−Ni complex inks through mixing selected Cu and Ni MOD precursors.^[^ ^32^ ^]^ These inks could be inkjet‐printed and sintered to form uniform Cu‐Ni‐core‐shell nanostructures. The different sintering temperatures of Cu and Ni MOD precursors were exploited to fabricate a desired core‐shell structure. During the sintering process Cu NPs were formed as the sintering temperature reached ≈150 °C and when the sintering temperature rose above 200 °C Ni MOD precursors started to decompose and form Ni metal‐protecting layers around earlier formed Cu NPs and clusters. However, the gap in sintering temperatures between Cu and Ni precursors limited the quality of printed patterns and the high sintering temperature of Ni MOD precursors restricted the application range of these inks.^[^ ^33^ ^]^

Building layered structures at the nano‐level or fabricating layered functional structures by assembling nano‐layers of units is a typical approach in nanoarchitectonics.^[^ ^34^ ^]^ The concept of layered nanoarchitectonics is broadly used in applied fields of catalysis,^[^ ^35^ ^]^ electronics,^[^ ^36^, ^37^, ^38^ ^]^ energy storage,^[^ ^39^ ^]^ medicine,^[^ ^40^, ^41^ ^]^ or biomedical applications^[^ ^42^ ^]^ Ji et al. (2023) built an interfacial lattice lock layer by electrodepositing Zn and Cu on a Zn electrode to optimize the long‐term stability of Zn‐metal rechargeable batteries.^[^ ^43^ ^]^ To overcome the difficulty of re‐stacking and sequential synthesis of conventional layer fabrication methods an approach to directly exfoliate high‐quality BiI3–BiI hetero‐structured nanosheets with alternating blocks from solution‐grown binary hetero‐crystals was developed.^[^ ^44^ ^]^ Metal dichalcogenide materials with vertical compositional gradient provide a platform for a variety of potential applications ranging from catalysis to quantum devices. Wang et al. constructed a metallic structure of AuCrS2 units grow on top of the first CrS2 monolayer by gold‐assisted chemical vapor deposition at 700 °C.^[^ ^45^ ^]^ Inkjet printing of hybrid MOD ink may provide an entirely different route from traditional layered nanoarchitectonics to fabricate metal layered structures with vertical compositional gradient with single‐step and low temperature.

As introduced above current hybrid inks could be used to fabricate fully‐mixed structures or core‐shell structures. Metallic patterns with vertical compositional layered distribution are very difficult to fabricate with one‐step printing.^[^ ^46^ ^]^ A metal‐based hybrid MOD ink normally includes multiple precursors with different decomposition temperatures which makes it challenging to meet certain rheological properties during the sintering process to obtain stable layered structures.^[^ ^47^ ^]^ In this study we proposed an ink design strategy for formulating metal‐based MOD hybrid inks which could be directly inkjet‐printed and stably sintered into controllable patterns with vertical compositional gradient. In previous works 2‐aminoethan‐1‐ol (EA) and aminopropan‐2‐ol (AP) have already been used to synthesize a series of Cu and Ag‐based precursors and could be made into inkjet printable MOD inks without any stabilizers or additives.^[^ ^28^, ^29^ ^]^ Based on the evaluation and analysis of Ag and Cu MOD precursors synthesised with EA and AP ligands and four Ag‐Cu hybrid MOD inks with different precursor combinations through density functional theory (DFT) computations and thermogravimetric analysis/mass spectrometry (TGA‐MS) measurements an inkjet printable Ag‐Cu‐based hybrid MOD ink was produced. The produced ink could fully decompose at the low temperature (<150 °C) and it could be sintered into designed metallic patterns on several types of flat substrates including glass cellulose fiber and polymer. When sintered under N_2_ the stable sintering process allowed the self‐regulating of in situ generated Ag and Cu particulates to form a two‐layered structure with vertical compositional gradient: a Cu layer on the top and an Ag layer beneath. The printed patterns showed high electric conductivity (σ > 10^6^ S m^−1^). When sintered under air Ag precursors decomposed into Ag nanoparticulate while Cu precursors decomposed into CuO and Cu nanoparticulate, the conductive patterns (σ > 10^4^ S m^−1^) were formed with a larger porous and more CuO‐dominant layer over the Ag‐Cu layer than the N_2_‐sintered pattern. The one‐step printed and air‐sintered patterns exhibited high electrocatalytic activity toward glucose in 0.1 M NaOH solution with 1 mM glucose.

## 2 Results and Discussion

**Figure 1:**
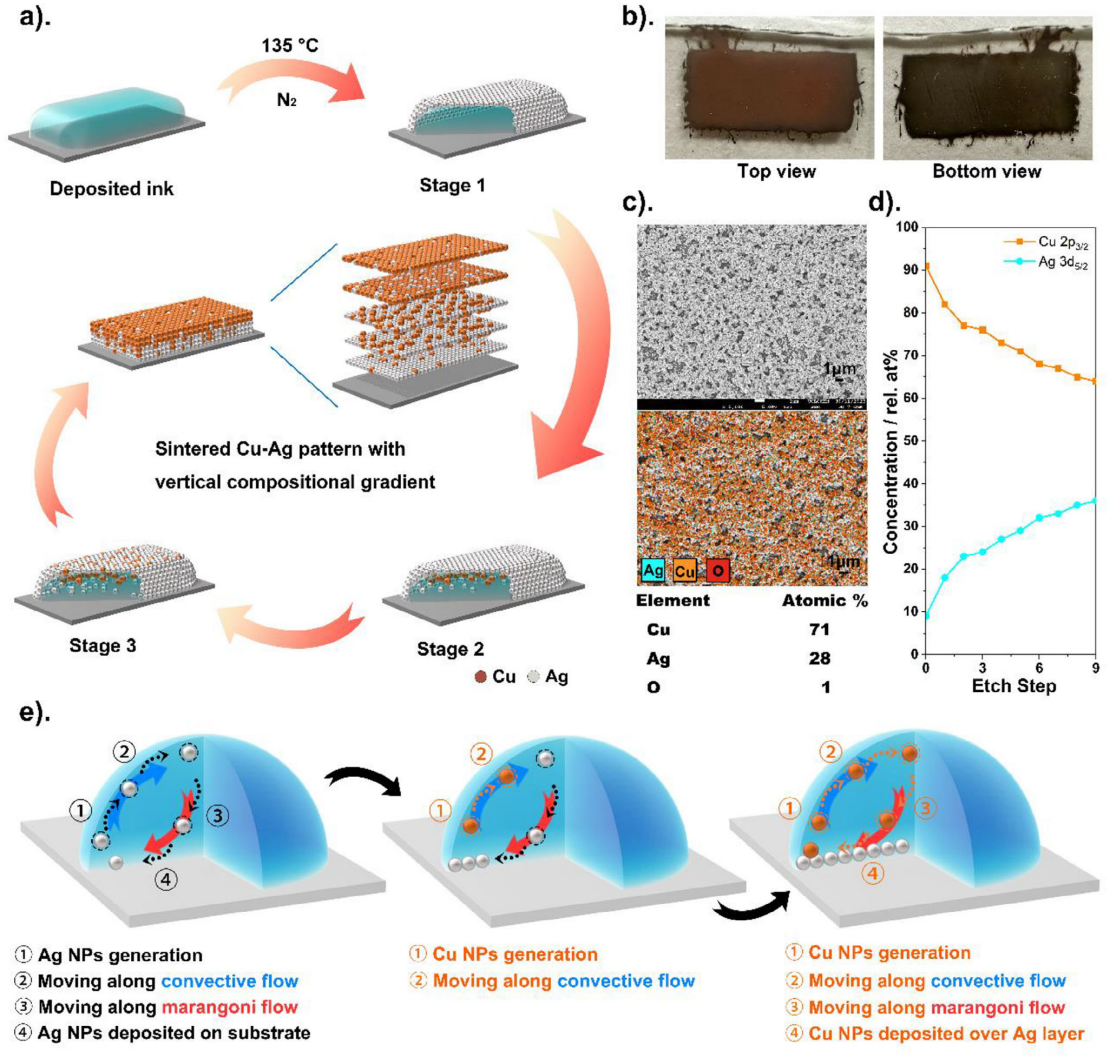
a) Schematic diagram explaining the formation process of Cu‐Ag pattern with vertical compositional gradient when the Ag‐Cu hybrid MOD ink is sintered under N_2_. b) Visual view of the sintered pattern from top and bottom. c) SEM image and EDS mapping of the pattern surface sintered in N_2_. d) XPS depth profile of patterns inkjet‐printed on a glass slide and sintered in N_2_. The Cu 2p3/2 and Ag 3d5/2 areas were fit and corrected by atomic sensitivity factors to obtain the respective elemental contributions. e) Schematic illustration of the hybrid MOD ink evolvement during sintering process.

In this study a unique metallic pattern with vertical Cu‐Ag compositional gradient was fabricated by single‐step inkjet printing and sintering at low temperature under N_2_. The schematic of the general sintering and structure formation process under N_2_ is illustrated in **Figure** **1**a. As shown in Video S1, Supporting Information when the printed Ag‐Cu hybrid MOD ink is sintered under N_2_ the Ag precursor decomposed as the volatile substances evaporate and Ag nano‐particulates start to be generated at the surface (on the interface with the air) from the edges to the center of the deposited pattern. These then float on the surface to form a “silver skin” due to the liquid tension of the deposited ink.^[^ ^48^ ^]^ Cu nanoparticulate generation follows Ag nano‐particulates moving upward as the Ag nano‐particulates sink downward. Eventually the two different layer structures that Cu atoms are dominantly distributed on the top layer are formed. As shown in Figure 1b and SI 10 the sintered patterns have a light reddish metallic hue on the top surface and a light silver color at the bottom surface (visually sighted through glass).

The fabricated samples were characterized by X‐ray diffraction (XRD) scanning electron microscopy (SEM) with energy‐dispersive X‐ray spectroscopy (EDS) and X‐ray photoelectron spectroscopy (XPS) depth profiles. Through film XRD analysis crystallized metallic silver and copper phases were detected on the sample surface (Figure S1, Supporting Information). As shown in Figure 1c SEM analysis showed that metallic nanoparticles with ≈100 to 500 nm in size were connected and stacked with each other and covered the surfaces. Pores attributed to gas generation during the sintering were observed on the surface. EDS analysis was carried out to determine elemental compositions on the printed patterns. Considering the energy of the applied electron beam energy (5 keV) the scan depth for the patterns is estimated to be < 200 nm.^[^ ^49^ ^]^ It was confirmed that the top surface of the printed pattern had more Cu (71 atomic%) than Ag (28 atomic%) explaining why the copper color appears on the top (Figure 1b). It was observed that the surface contains a very small amount of O which is attributed to the exposure of the sample to air during storage and transport. To determine the vertical elemental distribution of the fabricated samples XPS depth profile analysis was undertaken with a sputtering time of 30 s for the etch step. Survey spectra collected during the experiment (SI 2) show some levels of C N O and Na contamination most likely from sample preparation and prolonged storage of the samples in air. From the total area fits of the Cu 2p3/2 and Ag 3d5/2 core level spectra elemental profiles were derived. Before sputtering a surface, copper oxide layer is detected which is removed after the initial sputter step. Thereafter both Cu and Ag are predominantly present as metals. As shown in Figure 1d the surface of the sample was strongly dominated by Cu from the beginning (91 rel.at% Cu: 9 rel.at% Ag) and a fast subsequent increase in the Ag concentration to ≈36 ± 2 rel.at%.

For thermal sintering of deposited MOD inks two internal flows exist: convective flow from edge to outer rim and Marangoni flow from the outer rim to the down center of the droplet. These two flows caused the contraction effect^[^ ^50^ ^]^ to push the in situ generated nano‐particulates in the droplet shift to deposit from edge region to center. In this work the system was more complicated for the hybrid ink with Cu and Ag precursors. As the schematic illustration shown in Figure 1e ideally when sintering starts Ag nano‐particulates will generate earlier due to relatively higher redox potential the in situ generated Ag nano‐particulates will flow along the contraction effect and deposit on the substrate. The later in situ generated Cu nano‐particulates will follow the path of Ag nano‐particulates and deposit over Ag layer. In practice more Ag precursor decomposed at start and with the sintering process going on decomposition of Cu precursor was gradually taking advantage and eventually formed a vertical compositional gradient. This model requires the Ag and Cu precursors will not react with each other or restructure after mixing. These two precursors should have similar decomposition temperature and rate to avoid the formation of core‐shell structure and no extra solvents are required to make the hybrid ink printable to avoid the fully‐mixed structure and “coffee ring” issues.

**Figure 2:**
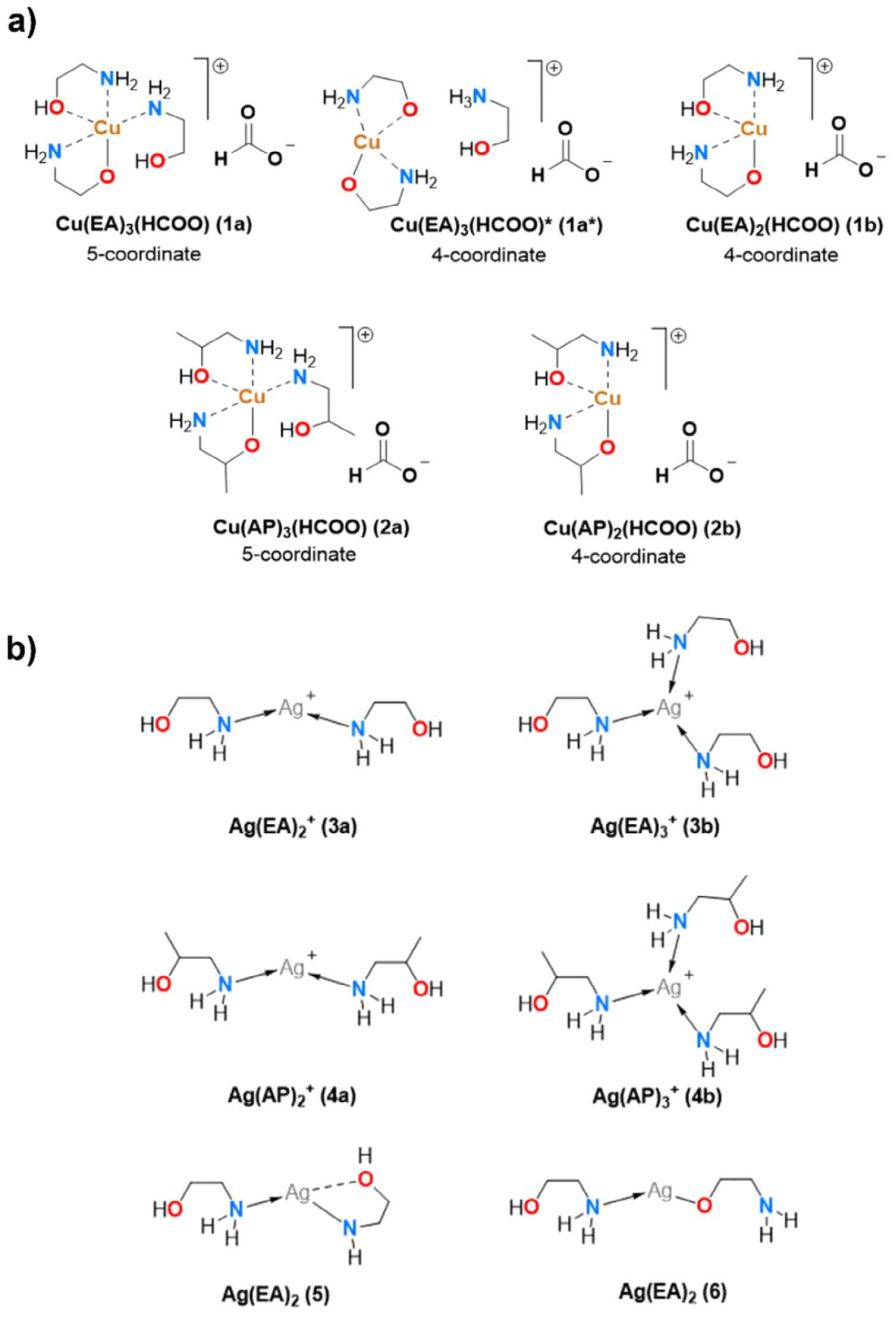
a) Structures of Cu‐EA (top row) and Cu‐AP (bottom row) precursors studied by DFT. b) Structures of Ag‐EA (top row) and Ag‐AP (middle row) precursors studied by DFT. Bottom row represents the two other (5 and 6) formulations for the Ag‐EA precursor considered in this study.

*Computational and Decomposition Studies*: Before the experimental formulation of Ag‐Cu hybrid MOD inks DFT computations were performed to gain more insight into the formulation of the Ag and Cu precursors. Previous computational studies conducted for a Cu‐based EA precursor ink (**Figure** **2**a) indicated that the preferred formulation is a 4‐coordinate complex 1a* instead of a 5‐coordinate one (1a) the energy difference of the two structures has been calculated to be small (ΔG < 3.3 kcal mol^−1^, ωB97X functional).^[^ ^51^ ^]^ These studies only concentrated on the arrangement and relative stability of three chelating EA‐ligands around Cu^2+^ cation and HCOO− anion. Now extending the studies further we performed DFT calculations (SI 3) using the PBE0 functional with Grimme's empirical dispersion correction (GD3BJ) and corrected the energies for solvent.^[^ ^52^, ^53^, ^54^ ^]^ Using this method and including the formate anion in the calculations the free energy difference of the two complex structures diminished to 2.3 kcal mol^−1^ in favor of the 4‐coordinate species 1a*. This species however includes two anionic EA‐ligands [H_2_NCH_2_CH_2_O]^−^ and one protonated [H_3_NCH_2_CH_2_OH]^+^. However, if the analogous calculations are done for a complex where there is one neutral and one anionic [H_2_NCH_2_CH_2_O]^−^ ligand around the Cu^2+^ ion (i.e. 1b in Figure 2a) the calculated free energy difference of the two coordination modes favors the 4‐coordinated species by 0.9 kcal mol^−1^ (ΔG_1b−1a_) and the free energy difference of structures 1a* and 1b is 3.1 kcal mol^−1^ in favor of 1b. Therefore, the rest of the calculations for the precursors incorporate only one anionic ligand component in the structures.

An analogous optimization and coordination study was conducted for the copper species with either two or three AP‐ligands surrounding the Cu^2+^‐cation and the 5‐coordinate structure 2a was found to be favored over the 4‐coordinate 2b by 0.5 kcal mol^−1^ which corresponds to the experimentally observed structure in the solid state.^[^ ^51^ ^]^ As a whole the Gibbs free energies for the formation of complexes 1 and 2 from Cu (II) formate and EA/AP were calculated to be exergonic. For the EA‐ligand the formation of 1b was calculated to be −17.4 kcal mol^−1^ whereas for 1a it was −16.5 kcal mol^−1^. In contrast the calculated free energies for the formation of Cu‐AP complexes were −20.2 (1a) and −19.7 kcal mol^−1^ (1b).

Experimentally the Ag‐precursors are made from silver(I) acetate and excess EA/AP with a 0.5 equivalent of formic acid. Thus, during the formulation of the precursor, it is possible to either have the acetate as a counter anion for Ag^+^ or deprotonation of the formic acid to produce formate anion and acetic acid (Figure 2b). The decomposition process of the precursor is thought to occur due to the reduction of Ag^+^ to metallic Ag by the formate. Hence the amount of added formic acid has to be controlled during the synthesis of the precursors. The DFT calculations showed that for both 3a and 4a the formation of acetic acid and a formate anion is equally more favourable (ΔG_3a_ = −2.8 kcal mol^−1^ and ΔG_4a_ = −2.8 kcal mol^−1^) than a combination of acetate and formic acid. The energy difference though is quite small and in experimental conditions it could likely have a mixture of the two anions as a sub‐stoichiometric amount of formic acid is present. The ligands EA and AP can also be deprotonated to form overall neutral complexes 5 and 6. However the formation of 5 was calculated to be endergonic by 13.5 kcal mol^−1^ and the formation of 6 was approximately thermoneutral (ΔG = −0.3 kcal mol^−1^) ruling out these possibilities in the experimental conditions.

DFT calculations to determine whether 3a/4a or 3b/4b is the more stable structure suggest that 3a and 4a are favored over 3b and 4b, respectively (ΔG_3a‐3b_ = −4.2 kcal mol^−1^ and ΔG_4a‐4b_ = −1.5 kcal mol^−1^ formate counter anion). Overall the Gibbs free energies for the formation of complexes 3 and 4 were calculated to be exergonic. For the EA‐ligand the formation of 3a was calculated to be −13.6 kcal mol^−1^ whereas for 3b it was −9.4 kcal mol^−1^. Similarly, the calculated free energies for the formation of Ag‐AP complexes were −15.0 and −13.6 kcal mol^−1^ for 4 a and b, respectively. Computationally the formation of all complexes 1–4 is energetically favored and the Gibbs free energies are strongly negative in all cases. This suggests that ligand scrambling upon mixing the copper and silver precursors to make the hybrid inks is unlikely which was also supported by FT‐IR analysis (SI 4).

**Figure 3:**
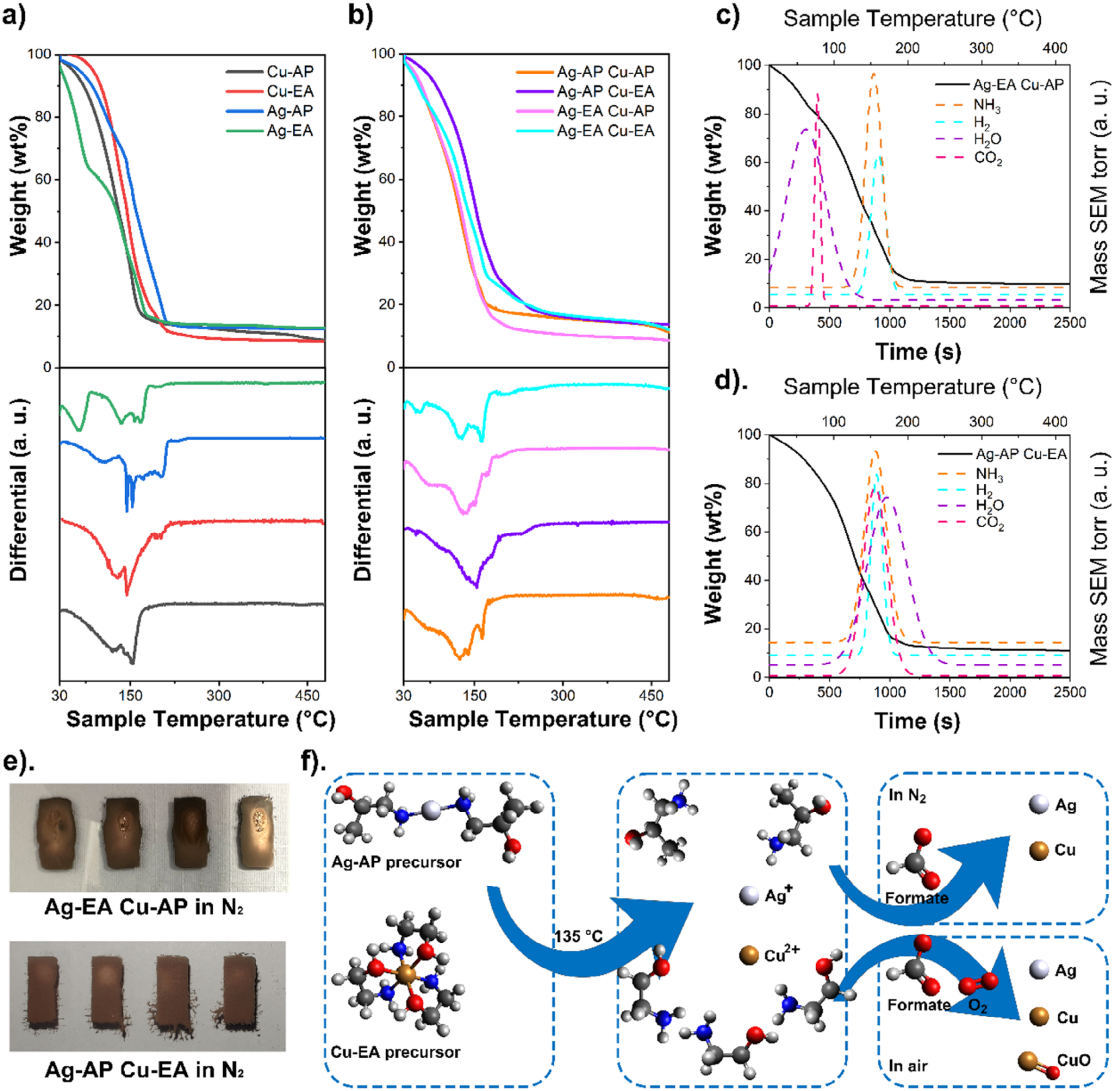
a) Overlapping TGA curves (top) and offset derivatives (bottom) of inks: Cu‐AP (black) Cu‐EA (red) Ag‐AP (blue) and Ag‐EA (green) MOD precursors. b) Overlapping TGA curves (top) and offset derivatives (bottom) of hybrid MOD inks consisting of: Ag‐AP with Cu‐AP (orange) Ag‐AP with Cu‐EA (purple) Ag‐EA with Cu‐AP (pink) and Ag‐EA with Cu‐EA (light blue). c) Gauss fit TGA‐MS curve of the hybrid MOD ink consisting of Ag‐EA and Cu‐AP precursors. d) Gauss fit TGA‐MS curve of the hybrid MOD ink consisting of Ag‐AP and Cu‐EA precursors. e) Inkjet‐printed patterns of Ag‐EA Cu‐AP precursor‐based ink and Ag‐AP Cu‐EA precursor‐based ink on the glass slide and sintered at 135 °C in N_2_. f) Schematic of decomposition reactions of the Cu‐EA and Ag‐AP precursors in the Ag‐Cu hybrid MOD ink during the sintering process in N_2_ and in the air.

Experimentally four Cu and Ag MOD precursors were synthesised with EA and AP ligands and in situ formulated into inks. Four Ag‐Cu hybrid MOD inks were formulated by mixing Cu and Ag MOD inks in this study. Ethanol was used to adjust the surface tension and viscosity of formulated inks for inkjet printing. The metallic contents and decomposition temperatures of four in situ formulated Cu and Ag inks (**Figure** **3**a) and four formulated Ag‐Cu hybrid MOD inks (Figure 3b) were evaluated by thermogravimetric analysis (TGA). The decomposition temperature is the lowest temperature at which the deposited ink can fully decompose into elemental metal at the fastest rate. In MOD inks the maximum metallic content is limited by the solubility of cations in the solution. Hence the evaporation of solvents will also cause the decomposition of the precursor indicating that the practical sintering temperature could be lower than the theoretical decomposition temperature taken from TGA. The practical sintering temperature also depends on the properties of substrates such as wettability or thermal sensitivity.^[^ ^17^, ^48^ ^]^ However in general a relatively high sintering temperature could shorten the sintering time form metallic films with higher conductivity and have better pattern quality control.

The in situ formulated Ag‐EA (green) and Ag‐AP MOD (blue) inks had a similar Ag content of 12.75 wt.% and 12.56% wt.%, respectively. The in situ formulated Cu‐EA MOD ink (red) was composed of 8.46 wt.% copper and the copper content of in situ formulated Cu‐AP MOD ink (black) was measured to be 8.25 wt.%.

The derivatives of TGA curves unraveled the decomposition processes of in situ formulated MOD precursor inks. For the Ag‐EA MOD ink (green) a distortion with a strong derivative peak was detected ≈63 °C which might be the evaporation of ethanol. Distortions appeared in the range from ≈84 to ≈180 °C with peaks at 135, 157, and 168 °C related to the decomposition of the precursor. For the Ag‐AP MOD ink (blue) a gentle distortion at ≈72 °C is due to ethanol evaporation. Distortions related to the decomposition were detected from ≈132 to ≈216 °C with two strong peaks at 144 and 153 °C. For the Cu‐EA MOD ink (red) no isolated distortion or peak related to the evaporation was detected but two peaks appeared at 127 and 144 °C. For the Cu‐AP MOD ink (black) two peaks appeared at 120 and 154 °C. Hence the formulated Ag‐EA and Ag‐AP inks have an onset of decomposition from 84 and 132 °C and they would theoretically decompose fully at 153 and 168 °C, respectively. The formulated Cu‐EA and Cu‐AP would theoretically decompose fully at 144 and 154 °C, respectively.

Following this preliminary assessment hybrid inks were then investigated: the formulated Ag‐Cu hybrid MOD inks consisting of Ag‐AP with Cu‐AP (orange) Ag‐AP with Cu‐EA (purple) Ag‐EA with Cu‐AP (pink) and Ag‐EA with Cu‐EA (light blue) consisted metallic content of 10.69 13.60 8.02 and 10.80 wt.%, respectively. The ink of Ag‐AP with Cu‐EA had the highest metallic content while the ink of Ag‐EA with Cu‐AP had the lowest metallic content because higher ethanol content is required to adjust the viscosity to make it printable (SI 5).

The decomposition process of these four hybrid MOD inks was analyzed using the derivatives of TGA curves. Multiple peaks appeared. For Ag‐EA with Cu‐EA peaks at 54 128 and 164 °C were observed on the derivative plots of Ag‐EA with Cu‐EA and Ag‐AP with Cu‐AP. For Ag‐AP with Cu‐AP peaks at 97 141 and 163 °C were confirmed. The multiple peaks on the derivative plot indicate a potentially unstable sintering process of hybrid MOD inks which might result in unexpected and undesired pattern structures.^[^ ^35^ ^]^ A gentle distortion at ≈77 °C and a peak at 137 °C appear on the derivative plot of Ag‐EA with Cu‐AP. For Ag‐AP with Cu‐EA only one peak at 154 °C was detected. Compared to the other two formulated hybrid MOD inks Ag‐EA with Cu‐AP and Ag‐AP with Cu‐EA were considered relatively more suitable for fabricating controllable printed structures.

To investigate further the decomposition processes of inks of Ag‐EA with Cu‐AP and Ag‐AP with Cu‐EA TGA‐MS measurements were carried out. The temperatures at which precursors start to decompose will be linked to the gas and vapor generations at different temperatures. In the TGA‐MS analysis of Ag‐EA with Cu‐AP (Figure 3c) CO_2_ water H_2_ and ‐NH_3_ fragments were detected. Water and CO_2_ generated by the decomposition of the precursor was negligible on the Gauss fit plot of mass spectrometry because of the much smaller amount formed compared to the water and CO_2_ detected in the ranges of ≈50 to ≈100 °C indicating the evaporation and decomposition of the ethanol in ink. H_2_ and ‐NH_3_ fragments were detected from ≈105 to ≈186 °C due to the precursor decomposition implying that the precursor could start to decompose at ≈105 °C. As shown in Figure 3d CO_2_ water H_2_ and ‐NH_3_ fragments generated by the precursor decomposition were detected in the TGA‐MS analysis of Ag‐AP with Cu‐EA. H_2_ and NH_3_ were generated from ≈114 to ≈191 °C indicating that the precursor could start to decompose at ≈114 °C. Hence in a practical sintering process the sintering temperature of Ag‐EA with Cu‐AP could range from 105 to 137 °C and the sintering temperature of Ag‐AP with Cu‐EA could range from 114 to 154 °C (≈134 °C in average). As shown in Figure 3e the extra‐added ethanol (SI 5) in Ag‐EA with Cu‐AP aggravated the “coffee ring” issue and resulted in poor pattern quality. Hence in this study an inkjet‐printable Ag‐Cu hybrid MOD ink was fabricated by combining Ag‐AP and Cu‐EA MOD precursors at a volume ratio of 1:1 (Cu: Ag content ratio in mole = 1.05:1). The practical sintering temperature was selected to be 135°°C based on the results of TGA analysis with the consideration of both sintering time and pattern quality. It was also expected that during the sintering under N_2_ the precursors would begin to decompose and reduce to elemental copper and silver by formate anions but copper (II) oxide would also form due to oxidation when sintering under air (Figure 3f).^[^ ^51^ ^]^

**Figure 4:**
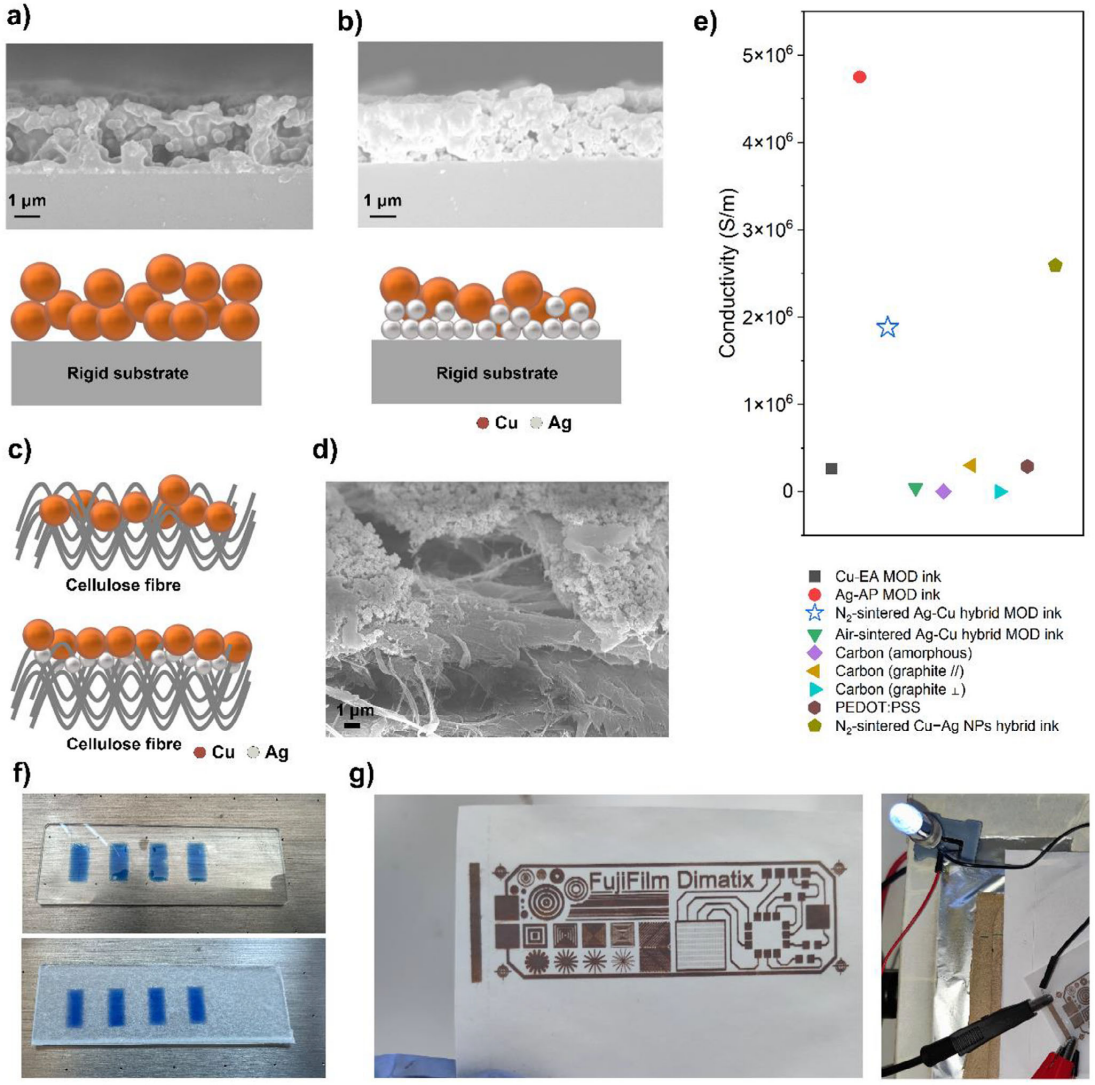
a) SEM image and schematic diagram of the N_2_‐sintered Cu‐EA ink pattern cross‐section on a glass slide. b) SEM image and schematic diagram of the N_2_‐sintered Ag‐Cu hybrid MOD ink pattern cross‐section on a glass slide. c) Schematic diagram of N_2_‐sintered (up) Cu‐EA ink pattern cross‐section and (down) Ag‐Cu hybrid MOD ink pattern cross‐section on cellulose fiber substrate. d) SEM image of the N_2_‐sintered Ag‐Cu hybrid MOD ink pattern cross‐section on cellulose fiber substrate. e) Electrical conductivity comparison of N_2_‐sintered Cu‐EA MOD,^[^ ^29^ ^]^ Ag‐AP MOD^[^ ^28^ ^]^ and Ag‐Cu hybrid MOD ink patterns (our work) air‐sintered Ag‐Cu hybrid MOD ink pattern (our work) carbon‐based materials (amorphous and graphite forms),^[^ ^41^ ^]^ PEDOT: PSS,^[^ ^42^, ^43^ ^]^ and N_2_‐sintered Cu‐Ag NPs hybrid ink.^[^ ^11^ ^]^ f) Inkjet‐printed ink on glass slide and cellulose fiber substrate. g) Conductive complex pattern fabricated with N_2_‐sintered Ag‐Cu hybrid MOD ink on cellulose fiber substrate.

**Electrical Conductivity Study**: For printed electronics the conductivity of sintered metallic film patterns is highly depending on packing density.^[^ ^44^ ^]^ The cross‐section images in **Figure** **4**a,b show that the N_2_‐sintered Cu‐EA ink pattern had large porous structures which could result in a reduced electrical conductivity whereas in the case of the Ag‐Cu hybrid MOD ink with the vertical compositional gradient structure small Ag nanoparticles (<100 nm in size) filled the gap between Cu nanoparticles (>500 nm in size) creating a higher packing density.^[^ ^28^, ^29^ ^]^ All N_2_‐sintered patterns on glass slides were conductive with an average conductivity of 1.88 ± 0.7 × 10^6^ S m^−1^ (SI 6). It was compared with the results of previous studies to validate the conductivity of the Ag‐Cu MOD hybrid ink pattern. As shown in Figure 4e the conductivity of the Ag‐Cu MOD hybrid ink pattern was lower than that of the Ag‐AP MOD ink pattern but much higher than Cu‐EA MOD ink and other widely used electrode materials for flexible electronics such as carbon‐based materials and PEDOT: PSS.^[^ ^55^, ^56^, ^57^ ^]^ In addition its sintering temperature is lower than that of the Cu‐Ag NPs hybrid ink which requires over 170°°C.^[^ ^58^ ^]^ For growing application scenarios substrates with complex surface morphologies such as cellulose fibers are widely used. The capillarity of textile to ink and large gaps among fibers make that multiple‐layer printing is required for traditional metallic inks to form conductive patterns with high resolution.^[^ ^59^ ^]^ In this work the formulated Ag‐Cu MOD hybrid ink can be inkjet printed on both rigid glass substrates and cellulose fiber substrates with excellent wettability performance as shown in Figure 4f. When sintered on cellulose fiber substrates the beneath Ag nanoparticles full‐filled the gaps via capillarity (Figure 4d) and support the upper Cu layer to form uniform high‐resolution patterns (Figure 4g) with average conductivity of 1.82 ± 0.2× 10^5^ S m^−1^.

**Figure 5:**
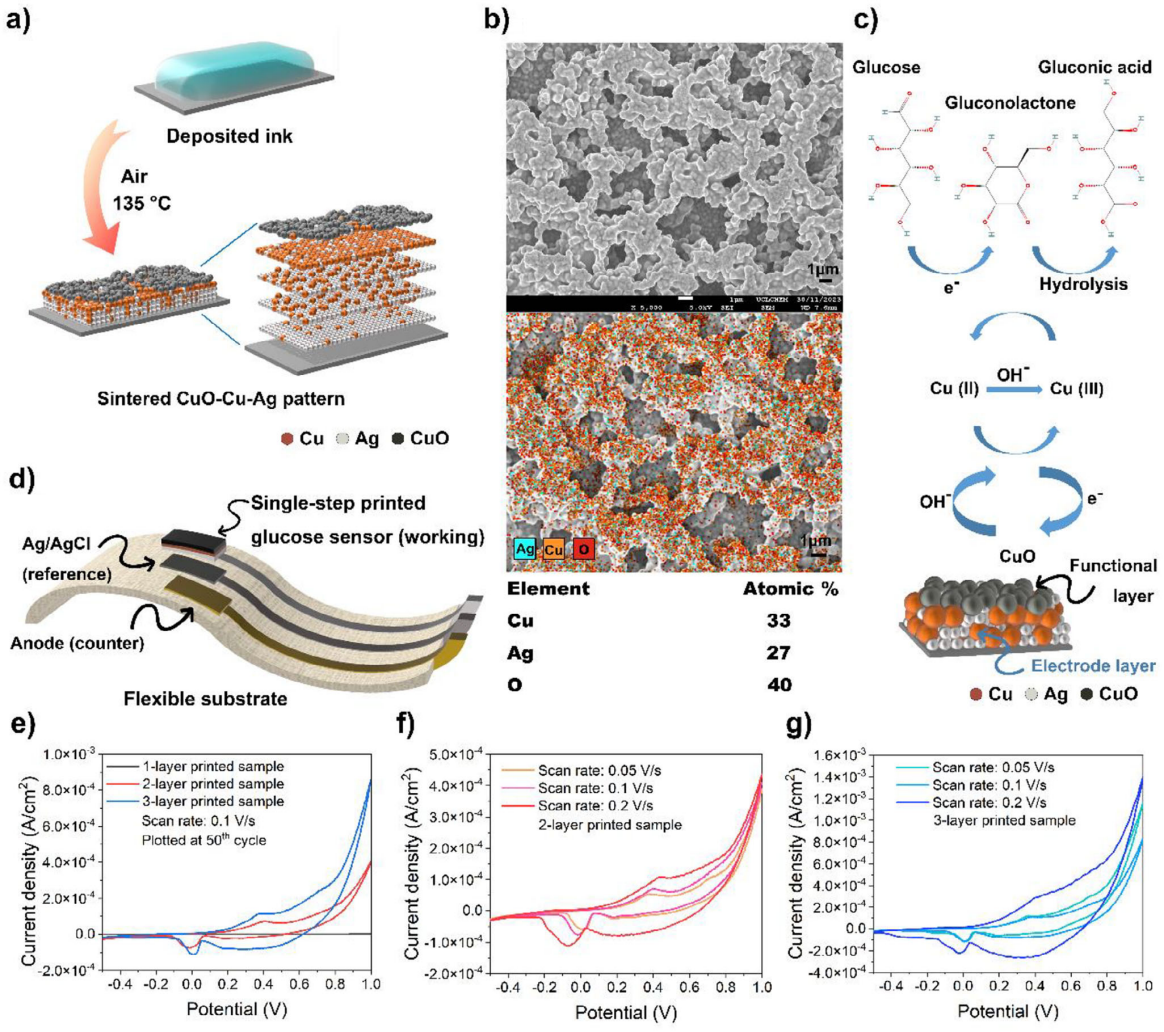
a) Schematic diagram explaining the formation process when the Ag‐Cu hybrid MOD ink is sintered in air. b) Schematic illustration of single‐step printed glucose sensor on the flexible substrate. c) SEM image and EDS mapping of the pattern surface sintered in air. d) Schematic diagram of the chemical reactions between CuO from the pattern surface and glucose in solution. e) Cyclic voltammograms (CVs) of the 1 2 and 3‐layer printed samples in 0.1 M NaOH solution with 1 mM glucose at a scan rate of 0.1 V s^−1^ for 50 cycles the 50^th^ cycle was plotted. CVs of f) 2 and g) 3‐layer printed samples in 0.1 M NaOH solution with 1 mM glucose for 50 cycles at the scan rates of 0.05 0.1 and 0.2 V s^−1^. The 30th cycle was plotted in each scan.

*Glucose Detection*: In the case of the deposited hybrid MOD ink sintered under air (**Figure** **5**a) a CuO layer could be dominantly produced on top because of the exposure to oxygen during the air‐sintering process. For the pattern sintered in air big pores ranging from 0.5 to 5 µm in diameter were observed across the surface. EDS analysis showed that apart from the Ag and Cu the surface had a large amount of O (40 atomic%) (Figure 5b). The big porous structure on the top surface was caused by the CuO clusters which results in a dark grey color.^[^ ^39^ ^]^ It is speculated that during the sintering in air the CuO nanoparticles were formed first because of oxygen exposure. This created a coating on the top of the ink preventing a further oxidation of the remaining Cu ink in the bulk and allowing Cu metal to form beneath this protective layer. This protective layer however has a detrimental effect on the morphology as it prevents gas release from inside to the outside of the surface during the sintering and resulted in porous and foam‐like surface morphologies. Through film XRD analysis the existence of CuO on the surface of the sample sintered in air was detected (Figure S2, Supporting Information). In addition, CuO's existence on of both N_2_ and air‐sintered samples was observed by XPS analysis (SI 2) and the peak for CuO was more intense on the sample sintered under air than that under N_2_. Copper(II) oxide (CuO) is a p‐type semiconductor with a narrow band gap of 1.2 eV. As shown in Figure 5c the hydroxyl‐ion adsorption and the semiconductive properties of CuO are proposed to be the mechanisms that render CuO electrocatalytically active facilitating the direct oxidation of glucose.^[^ ^60^ ^]^ However in recent researches a new hypothesis of electro‐oxidation mechanism of glucose by CuO is proposed. There might be no Cu (II) /Cu (III) transition happens. But with the presence of glucose in alkaline solution the electron transfer is facilitated and involves the transfer of electrons from the adsorbed hydroxyl ions to the CuO semiconductor film.^[^ ^61^ ^]^ It is determined that the semiconductor aspect of CuO plays key role in glucose detection. The beneath conductive layer (4.12 ± 1.5 × 10^4^ S m^−1^) strongly connected with CuO layer with the structure of vertical compositional gradient. Thus, it would be possible to directly fabricate a non‐enzymatic glucose sensor (Figure 5d) including both functional layer and conductive layer through the single‐step inkjet printing of the hybrid ink and air sintering process. Precise patterns were prepared and air‐sintered at 135°°C for cyclic voltammetry (CV) tests in 0.1 M NaOH solution with 1 mM glucose. The samples acted as the cathode (working) with an Ag/AgCl reference electrode and a platinum anode (counter). Each printed layer was set to have the same printing resolution indicating that the amount of ink deposited on a certain area was accurately controlled. The total ink deposition amount is controlled through different settings of printing layers in printer software.

As shown in Figure 5e for the 1‐layer printed sample there were no peaks corresponding to the Cu (II)/Cu (III) oxidation and reduction was detected during 50‐cycled CV tests at a scan rate of 0.1 V s^−1^.^[^ ^62^ ^]^ Clear oxidation peaks were detected at 0.38 V for 2 and 3‐layer printed samples. In addition, 2‐layer printed samples were more uniform with fewer defects and bubbles indicating better surface morphology than 3‐layer printed samples (SI 7). 2‐layer (Figure 5f) and 3‐layer (Figure 5g) printed samples were tested at scan rates of 0.05 0.1 and 0.2 V s^−1^ to ensure the reaction existed and the redox peaks to glucose were detected in all experimental conditions. For the 2‐layer printed sample when the scan rate rose to 0.2 V s^−1^ peaks shifted from 0 to – 0.07 V and from 0.38 to 0.44 V. For the 3‐layer printed sample peaks shifted from 0 to – 0.02 V and from 0.38 to 0.41 V indicating that the 2‐layer sample was more sensitive to scan rate change. After continuous CV measurements for 2 h 2‐layer printed samples showed outstanding adhesion to the substrate and still maintained electrochemical properties while the 3‐layer printed samples were fragile and almost completely delaminated after CV running indicating that the 2‐layer printed sample is superior (SI 7). Whilst glucose sensing is a promising approach tackling the challenges in areas of health it is a topic that has been covered in more detail elsewhere.^[^ ^63^, ^64^ ^]^ This experiment indicates that our simple low‐cost scalable strategy can simplify multi‐step fabrication methods to produce glucose sensors with an inbuilt silver electrode. Furthermore, this hybrid MOD precursor for developing electronic materials could be applied to flexible materials due to its low sintering temperature.

## 3 Conclusion 

In this work DFT structural studies were paired with thermal decomposition data for MOD ink formulation. DFT supported experimental evidence for the optimal coordination of amino‐alcohol ligands around the copper and silver centers which directly influences the decomposition profile. Regarding the formation of hybrid Ag‐Cu inks DFT showed that the Gibbs free energies of the individual Ag and Cu MOD precursors are strongly negative in all cases suggesting that ligand scrambling upon mixing the precursors to make the hybrid inks is unlikely. It was confirmed that among tested MOD inks Ag‐Cu hybrid MOD ink formed from silver with the larger branched amino‐alcohol (AP) and copper with the smallest steric profile ligand (EA) could be a superior inkjet‐printable ink. When the single hybrid MOD ink which was printed out on substrates sintered in N_2_ or the air at 135°°C two different self‐regulated structures with vertical compositional gradient in which Cu is dominantly distributed on the top layer were produced. Compared to that of the N_2_‐sintered sample a bigger porous structure on the top layer of the air‐sintered sample was produced and Cu and O distribution on the layer was higher indicating that CuO is more dominant distributed on it. In electrical conductivity measurement the N_2_‐sintered sample showed high conductivity on both rigid smooth substrates and cellulose fiber substrates. Its conductivity was even more prominent than Cu‐EA MOD ink carbon‐based materials and PEDOT: PSS. The air‐sintered Ag‐Cu hybrid sample showed the feasibility of glucose sensor application through single‐step printing which had high electrocatalytic activity against glucose and was stable over 2 h continuous measurement. It is expected that the Ag‐Cu hybrid MOD ink printable onto various substrates such as paper glass and plastic can be applied to the large‐scale production of low‐cost plastic or paper‐based flexible electronics. This research could provide a more useful approach to developing MOD ink formulations which can be applied to various electronics.

## 4 Experimental Section

### Materials

Silver (I) acetate (anhydrous) (99 wt. %) and copper (II) formate tetrahydrate (97% purity) were purchased from Alfa Aesar. 1‐aminopropan‐2‐ol (AP) (93 vol.%) and 2‐aminoethan‐1‐ol (EA) (≥99 vol.%) were supplied and purchased from Sigma Aldrich. Formic acid (≥95 vol.%) was purchased from Sigma Aldrich. Except for the anhydrous copper (II) formate all solvents and reagents for the ink formulation were used directly without any additional purification or filtrations. Anhydrous copper (II) formate was obtained by dehydration of copper (II) formate tetrahydrate under vacuum between 70 and 80°°C using Schlenk techniques. Fourier transform infrared (FT‐IR) spectroscopy was used to confirm the absence of water in the samples.

### In Situ formulation of Cu MOD Precursor

The synthesis of the Cu MOD precursor was amended from previous work.^[^ ^51^ ^]^ The mixing process was operated in an ice bath with a temperature of 0 to 3°°C. Anhydrous copper (II) formate (1 g 6.5 mmol) was vortex‐mixed with ethanol (1 mL 0.8 g) for 30 s and formed a dark blue slurry. 2‐Aminoethan‐1‐ol (4 mL 4.04 g 65 mmol) and 1‐Aminopropan‐2‐ol (4 mL 3.89 g 52.4 mmol) were vortex‐mixed with the slurry, respectively. The solutions were kept at room temperature overnight with continuous stirring. After that a clear blue Cu‐EA precursor ink was produced by filtering through a 200 nm syringe filter. For Cu‐AP precursor ink additional ethanol (1 mL 0.8 g) was added to the solution and vial containing the solution sealed and wrapped using aluminium foil. The solution was kept at room temperature overnight with continuous stirring. After that it was filtered through a 200 nm syringe filter to produce a clear Cu‐AP precursor ink.

### In Situ formulation of Ag MOD Precursor

The mixing process was operated in an ice bath with a temperature of 0 to 3°°C. Silver (I) acetate (0.5 g 2.96 mmol) was vortex‐mixed with ethanol (1 mL 0.8 g) for 30 s and formed a white homogeneous suspension. 2‐Aminoethan‐1‐ol (1 mL 0.973 g 16.6 mmol) and 1‐aminopropan‐2‐ol (1 mL 0.751 g 13.1 mmol) were vortex‐mixed with the suspension for 120 s, respectively resulting in a translucent light brown solution with a small amount of dispersed black precipitates. Formic acid (0.05 mL 0.061 g 1.3 mmol) was added dropwise and the solutions were vortex‐mixed after each dropwise. The vial containing the solution was sealed and wrapped using aluminium foil. The solution was kept at room temperature overnight with continuous stirring. After that it was filtered through a 200 nm syringe filter to produce a clear and transparent Ag‐EA and Ag‐AP precursor.

### Formulation of Ag‐Cu Hybrid MOD Inks

Four Ag‐Cu Hybrid MOD inks were synthesised and evaluated in this study: Cu‐EA precursor with Ag‐AP precursor Cu‐EA with Ag‐EA Cu‐AP with Ag‐EA and Cu‐AP with Ag‐AP. The calculated mole ratio of Cu and Ag content in the synthesised Ag‐Cu hybrid MOD ink was 1.05:1.

Except for Cu‐AP with Ag‐EA 1 mL of Cu MOD precursor was vortex‐mixed with 1 mL of Ag MOD precursor in all Ag‐Cu Hybrid MOD inks. The vial containing the mixture was sealed and wrapped using aluminium foil. The solution was kept at room temperature overnight with continuous stirring. After that it was filtered through a 200 nm syringe filter to produce a clear Ag‐Cu ink. For Cu‐AP with Ag‐EA the addition of ethanol (1 mL 0.8 g) was required for stir‐mixing.

### Preparation of Printed Metallic Patterns on Glass Slides PCBM Substrates and Cellulose Fiber Substrates

Samples were prepared by inkjet printing and thermally sintered in N_2_ and in the air, respectively. The designed patterns were inkjet printed with high printing resolution (2540 DPI) and thermally sintered at 135°°C in N_2_ or in the air for 1 h to ensure the printed samples were fully dried.

### Characterization—Fourier‐Transform Infrared Spectroscopy

Spectra in the range of 400 to 4000 cm^−1^ were recorded on Bruker ALPHA II FT‐IR with KBr pellets.

### Grazing Incidence X‐ray Diffraction

Films were measured with an Empyrean X‐ray diffractometer using glancing incident radiation at a beam angle of 1 ° with Cu K_α_ radiation (K_α1_ = 1.54056 Å K_α2_ = 1.54439 Å) and data were collected in the range of 10 ° < 2θ < 80 ° 0.05 ° step with 0.5 s/step.

### Thermogravimetric Analysis

Cu and Ag contents within the ink were measured using PerkinElmer STA 6000. 65 mg samples were placed in Al_2_O_3_ pans and heated from 30°°C up to a maximum of 500°°C at a heating rate of 10°°C min^−1^.

### Thermogravimetric Analysis/ Mass Spectrometry

Decomposition processes of hybrid inks were analyzed using Pyris 1 TGA (Perkin Elmer & Hiden MS HPR 20). The sample was placed and heated starting from 30°°C holding for 5 min then heating to 500°°C at 10°°C min^−1^ in a 40 psi Ar atmosphere. MS scanned from mass 0.1 to 100 as soon as the heating process in TGA started.

### Scanning Electron Microscopy

The surface morphology of films was imaged and analyzed with Jeol JSM 6701 FEG‐SEM at an accelerating voltage of 5 kV. The thickness of the printed film was also measured by SEM.

### Energy‐Dispersive X‐ray Spectroscopy

Samples in SEM were also measured with an accelerating voltage of 20 kV and analyzed by the Oxford Instrument EDS system.

### X‐ray Photoelectron Spectroscopy

XPS measurements were performed on a Thermo NEXSA photoelectron spectrometer at the EPSRC National XPS Service Didcot UK. A 400 µm spot size and a flood gun for charge compensation were used throughout. Survey and core level spectra were collected with pass energies of 200 and 40 eV, respectively. All samples were mounted using conducting carbon tape. Sputter depth profiles involved using an Ar sputter gun at 2 keV with each sputter step lasting 30 s.

### Droplet Surface Tension Measurements

The surface tension of the synthesised hybrid ink was measured with the KRUSS Drop Shape Analyzer. The needle diameter was 1.250 mm and the camera angle was set to 2°

### Electrical Conductivity Measurements and Calculations

The electric conductivity of the printed sample was measured through two probe resistance measurements with PeakTech Digital multimeter 4000 and calculated based on the specimen thickness from SEM measurement. For each sample printed on both glass slides and sulfuric paper substrates four samples were measured for the calculation of the average conductivity

### Glucose Sensing Test

Cyclic voltammetry (CV) tests were run for samples in 0.1 M NaOH solution with 1 mM glucose. The prepared sample was acting as the cathode (working) with an AgCl reference electrode and a platinum anode (counter). For each sample three scan rates were tested (0.05 0.1 and 0.2 V s^−1^) with 50 cycles for each scan rate. The active area (the area immersed in solution) for each sample is around 0.7 cm ×3.5 cm.

## Conflict of Interest

The authors declare no conflict of interest.

## Author Contributions

Ye Zhou (conceptualization data curation formal analysis investigation – ink synthesis and inkjet printing writing of original draft – lead contribution) Petra Vasko (formal analysis investigation – DFT calculation – supporting contribution) Yujiang Zhu (formal analysis investigation – glucose sensor test – supporting contribution) Jingyan Wang (data curation formal analysis – supporting contribution) Curran Kalha (supervision writing of review and editing – supporting contribution Anna Regoutz (XPS data analysis writing review and editing – support contribution) Adham Hashibon (review – support contribution) Yanlong Tai(manuscript review and editing support contribution) Gi Byoung Hwang (manuscript writing review and editing support contribution) Caroline E. Knapp (conceptualization funding acquisition project administration writing – original draft writing – review & editing – lead contribution). The manuscript was written through the contributions of all authors. All authors have given approval to the final version of the manuscript.

## Supporting information



Supporting Information

Supplemental Video 1

Supplemental Video 2

## Data Availability

The data that support the findings of this study are available from the corresponding author upon reasonable request.
