# Supplementary material for: Low‐Temperature Single‐Step Inkjet‐Printed Metallic Patterns With Self‐Regulated Vertical Compositional Gradient
Source: Small Methods. 2025 May 15;9(7):2401371. doi: 10.1002/smtd.202401371 (PMC12285616; doi:10.1002/smtd.202401371)
Supplement: Supplementary file 1 — Supporting Information [file SMTD-9-2401371-s002.docx]

Supplementary Information

Low-temperature, single-step inkjet-printed metallic patterns with self-regulated vertical compositional gradient

Ye Zhou,^[a][d]^ Petra Vasko,^[b]^ Yujiang Zhu,^[a]^ Jingyan Wang, ^[a]^ Curran Kalha,^[a]^ Anna Regoutz,^[a][e]^ Adham Hashibon,^[c]^ Yanlong Tai*,^[d]^ Gi Byoung Hwang*, ^[a]^ Caroline E. Knapp*^[a]^

[a] Dr Y. Zhou, Mr Y. Zhu, Ms J. Wang, Dr C. Kalha, Dr A. Regoutz, Dr G. B. Hwang, Dr C. E. Knapp, Department of Chemistry, University College London, 20 Gordon Street, London WC1H 0AJ, UK.

[b] Dr P. Vasko, Department of Chemistry, A. I. Virtasen aukio 1, P.O. Box 55, 00014 University of Helsinki, Finland

[c] Prof. A. Hashibon, Institute of Material Discovery, UCL East, Marshgate, 7 Sidings Street, London E20 2AE, UK

[d] Shenzhen Institutes of Advanced Technology, Chinese Academy of Sciences, Shenzhen 518055, China

[e] Department of Chemistry, University of Oxford, Inorganic Chemistry Laboratory, Oxford OX1 3QR, UK

E-mail: [caroline.knapp@ucl.ac.uk](mailto:caroline.knapp@ucl.ac.uk)

**SI 1. XRD analysis**


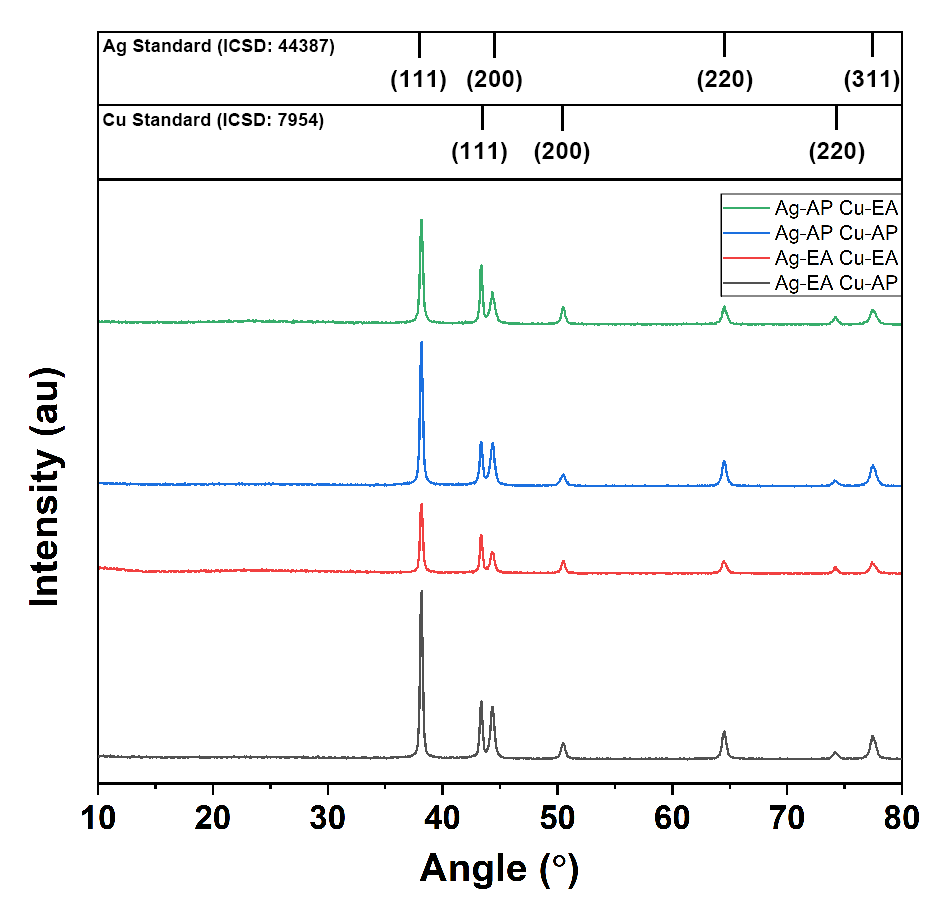


**Figure S1**. Grazing Incidence X-ray diffraction (XRD) patterns of formulated hybrid MOD inks sintered in N_2_ at 135 °C. Well crystallized metallic silver and copper phases were detected in all the samples.


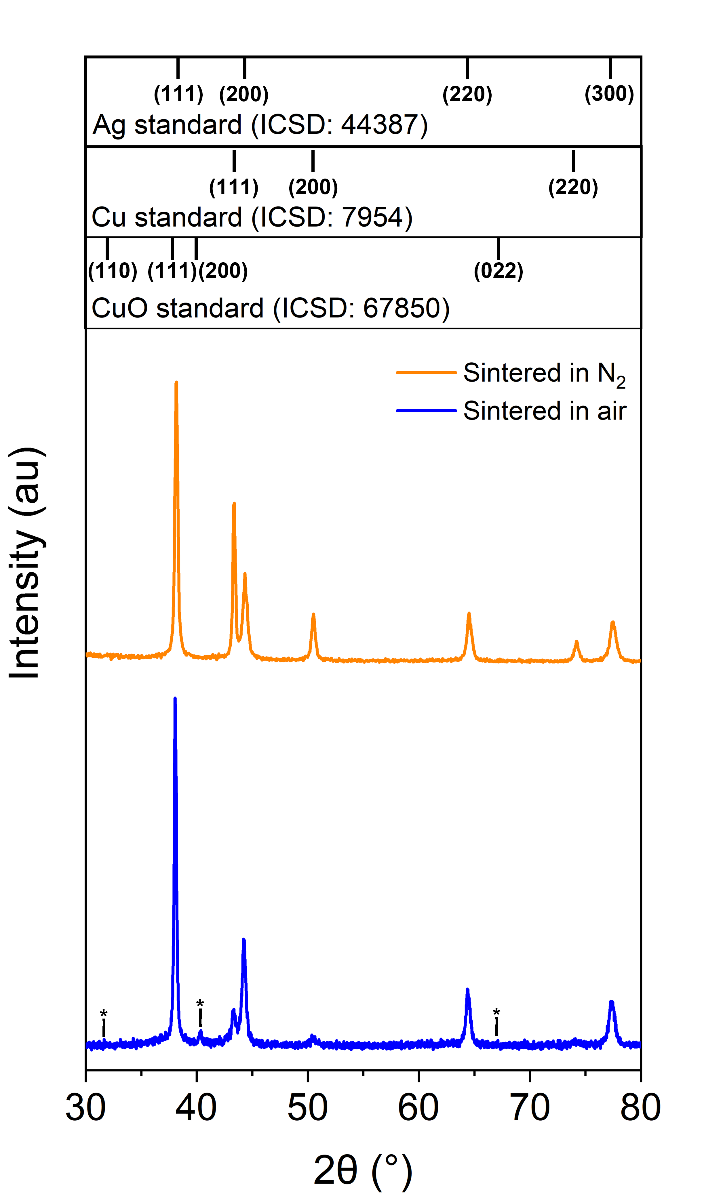


**Figure S2.** Grazing Incidence XRD patterns of the Ag-Cu hybrid MOD ink sintered in N_2_ and air at 135 °C, asterisks denote peaks from CuO.

**SI 2. XPS Survey**


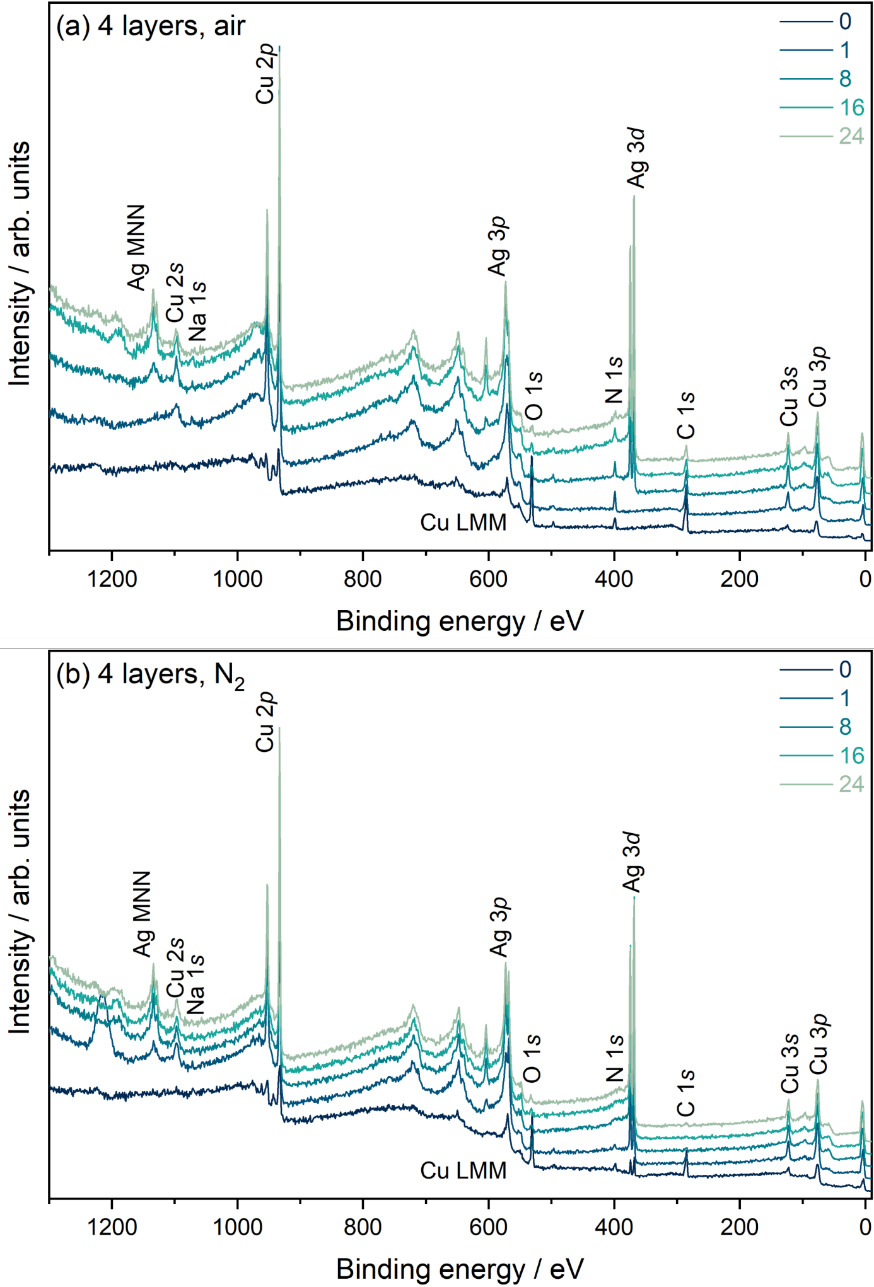


**Figure. S3:** Selected XPS survey spectra for films sintered in (a) air and (b) N_2_ across the depth profile. All major core and Auger-Meitner lines are identified. The numbers in the legends represent the etch step.

**
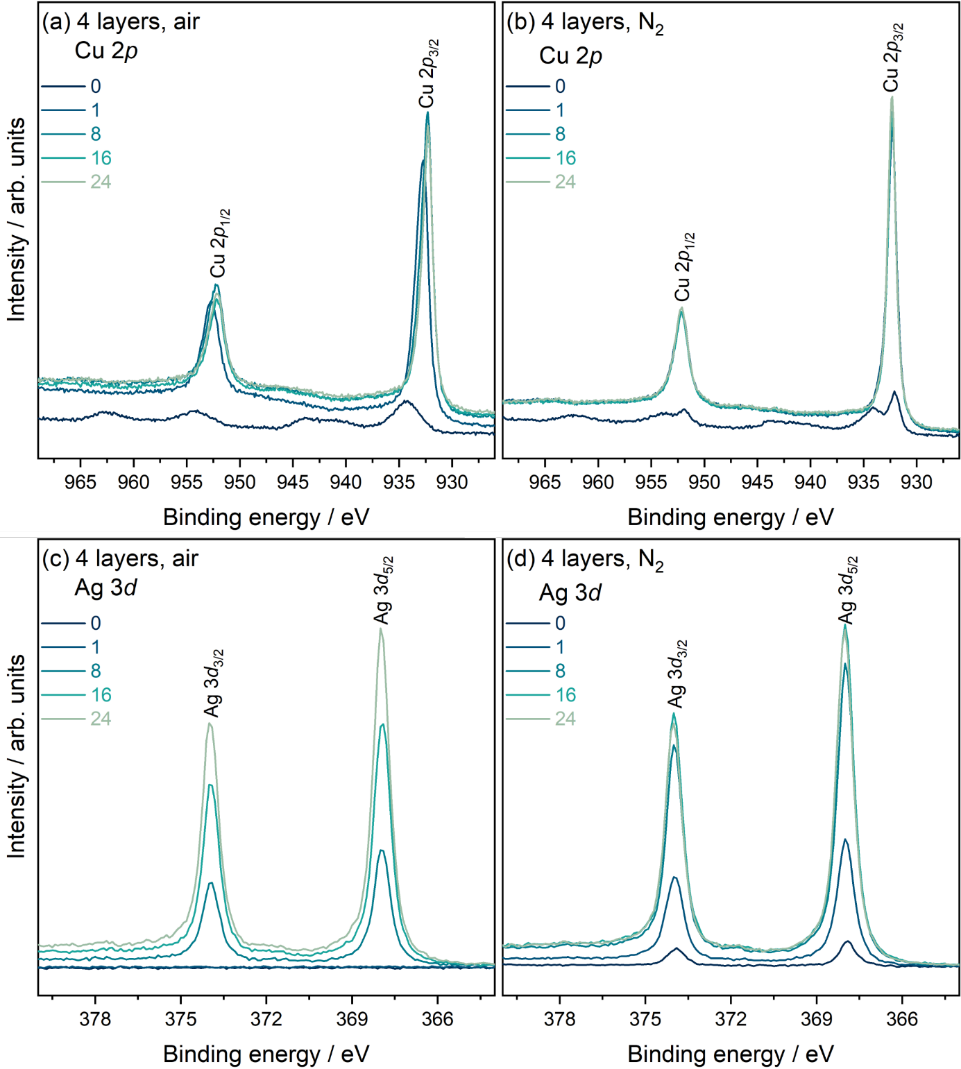
**

**Figure S4**. Selected XPS core level spectra for films sintered in (a) and (c) air and (b) and (d) N_2_ across the depth profile, including (a) and (b) Cu 2*p* and (c) and (d) Ag 3*d*. The numbers in the legends represent the etch step.

**SI 3. Computational details**

All computational work reported here was carried out at the density functional theory (DFT) level, using Gaussian16 (Revision C.01).^1^ The exchange-correlation functional PBE1PBE^2–4^ was employed in conjunction with the Def2-SVP^5,6^ basis set, Grimme's empirical dispersion correction (GD3BJ)^7^ and an ultrafine integration grid for the optimisation and frequency calculations. For silver, an external effective core potential (ECP) was applied.^8^ Single-point calculations were then performed at the PBE1PBE-GD3BJ/Def2-TZVP(PCM, solvent=ethanol)^9^ level. The given Gibbs free energies correspond to these corrected single-point energies at 298 K. The nature of the stationary points (minima) was confirmed by full frequency calculations at the Def2-SVP level and are characterized by zero imaginary frequency.

**SI 4. IR of formulated Ag MOD ink, Cu MOD ink, and Ag-Cu hybrid ink**

As shown in Figure S1, the transmittance peaks at 3352 and 3287 cm^-1^ could be assigned to the asymmetric and symmetric stretch of the NH_2_ group. The peaks at 2963, 2924, and 2861 cm^-1^ were assigned to CH_3_, CH_2_ and CH groups. A coupling of C-N stretching and in-plane bending of NH groups were indicated by the peaks of 1596 and 1460 cm^-1^, respectively. The C-O and O-H stretching vibrations were observed in a range of 1371 to 1053 cm^-1^. The spectra of Ag-Cu hybrid ink included all the peaks of Ag MOD ink and Cu MOD ink, indicating that ligand scrambling was unlikely to happen when mixing Ag MOD ink with Cu MOD ink.


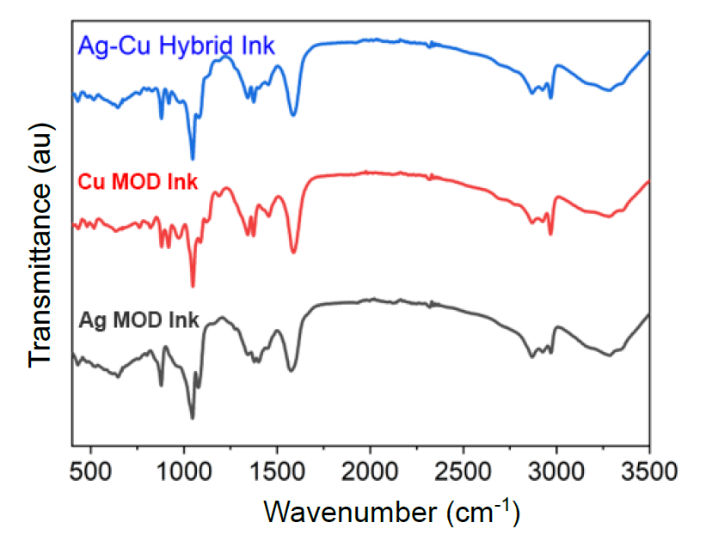


**Figure S5**. The FT-IR spectra of Ag MOD ink (Ag-AP MOD ink), Cu MOD ink (Cu-EA MOD ink), and Ag-Cu hybrid ink.

**SI 5. Pictures of formulated hybrid ink of Ag-EA with Cu-AP**


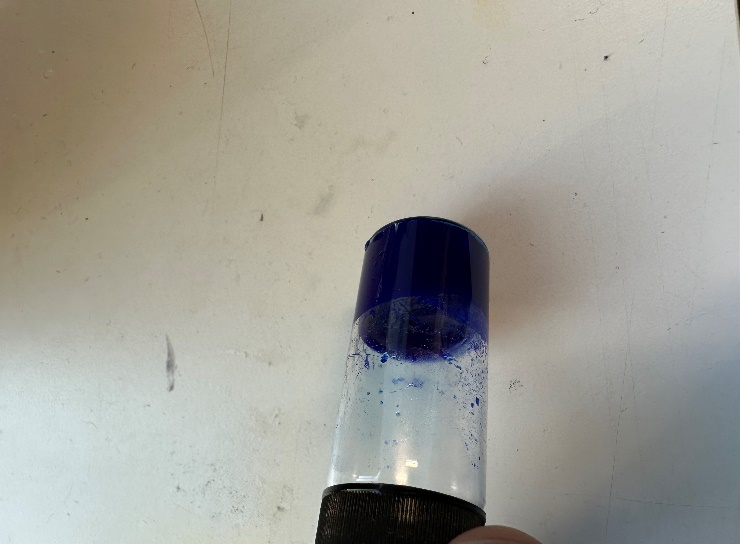

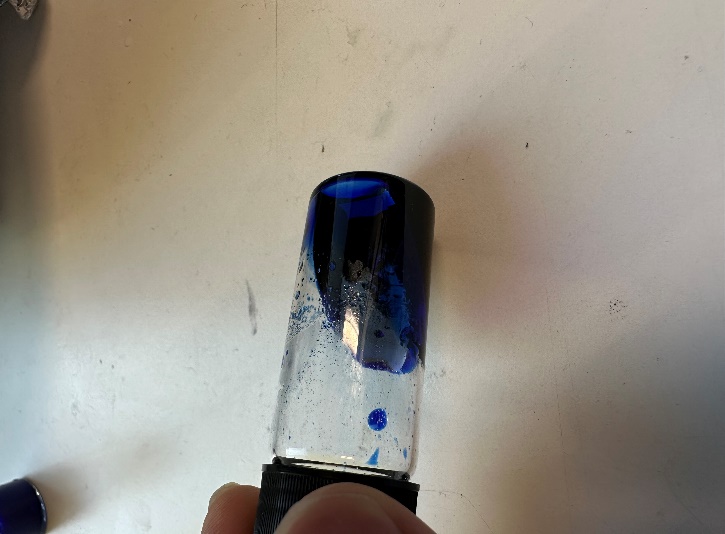


**(a)**

**(b)**

**Figure S6**. Formulated hybrid MOD ink of Ag-EA with Cu-AP (a) without extra ethanol and (b) with extra ethanol.

**SI 6. Electrical conductivity measurements and calculations**

In this study, formulated hybrid MOD ink of Ag-AP and Cu-EA was printed on glass and sulfuric paper substrates and sintered at 135 °C in N_2_ or in the air. The electric conductivity of the printed sample was measured through two probe resistance measurements with PeakTech Digital multimeter 4000 and calculated based on the specimen thickness from SEM measurement.

The electrical resistivity can be calculated by the equation:

$\rho=R\frac{A}{l}$ Eqn. 1

$$R is the electrical resistance of a uniform specimen of certain material$$

$$l is the length of the specimen$$

$$A is the cross-section area of the specimen$$

$Conductivity \sigma=\frac{1}{\rho}$ Eqn. 2

The dimensions of each specimen were practically measured but not only based on the designed rectangle pattern. The cross-section areas were calculated based on the specimen thickness from SEM measurement.

**Table S1.** Conductivity measurements and calculations

| Substrate | Sintering condition  (gas) | Temperature  [°C] | Resistance  [Ω] | Length  [m] | Cross-section Area  [m^2^] | Conductivity [S/m] | Average Conductivity  [S/m] |
| --- | --- | --- | --- | --- | --- | --- | --- |
| Glass | Air | 135 | 38.6 | 1.E-02 | 1.E-08 | 3.11E+04 | 4.12±1.5E+04 |
| Glass | Air | 135 | 40.5 | 1.E-02 | 1.E-08 | 2.96E+04 |  |
| Glass | Air | 135 | 19.9 | 1.E-02 | 1.E-08 | 6.03E+04 |  |
| Glass | Air | 135 | 27.4 | 1.E-02 | 1.E-08 | 4.38E+04 |  |
| Glass | N_2_ | 135 | 0.7 | 1.E-02 | 1.E-08 | 1.71E+06 | 1.88±0.7E+06 |
| Glass | N_2_ | 135 | 1.2 | 1.E-02 | 1.E-08 | 1.00E+06 |  |
| Glass | N_2_ | 135 | 0.5 | 1.E-02 | 1.E-08 | 2.40E+06 |  |
| Glass | N_2_ | 135 | 0.5 | 1.E-02 | 1.E-08 | 2.40E+06 |  |
| Paper | Air | 135 | 105.2 | 1.E-02 | 1.E-08 | 1.14E+04 | 3.29±3.9E+04 |
| Paper | Air | 135 | 115.4 | 1.E-02 | 1.E-08 | 1.04E+04 |  |
| Paper | Air | 135 | 56.9 | 1.E-02 | 1.E-08 | 2.11E+04 |  |
| Paper | Air | 135 | 13.5 | 1.E-02 | 1.E-08 | 8.89E+04 |  |
| Paper | N_2_ | 135 | 3.6 | 1.E-02 | 1.E-08 | 3.33E+05 | 1.82±0.2E+05 |
| Paper | N_2_ | 135 | 6.5 | 1.E-02 | 1.E-08 | 1.85E+05 |  |
| Paper | N_2_ | 135 | 10.4 | 1.E-02 | 1.E-08 | 1.15E+05 |  |
| Paper | N_2_ | 135 | 12.6 | 1.E-02 | 1.E-08 | 9.52E+04 |  |

**SI 7. Glucose sensing test**


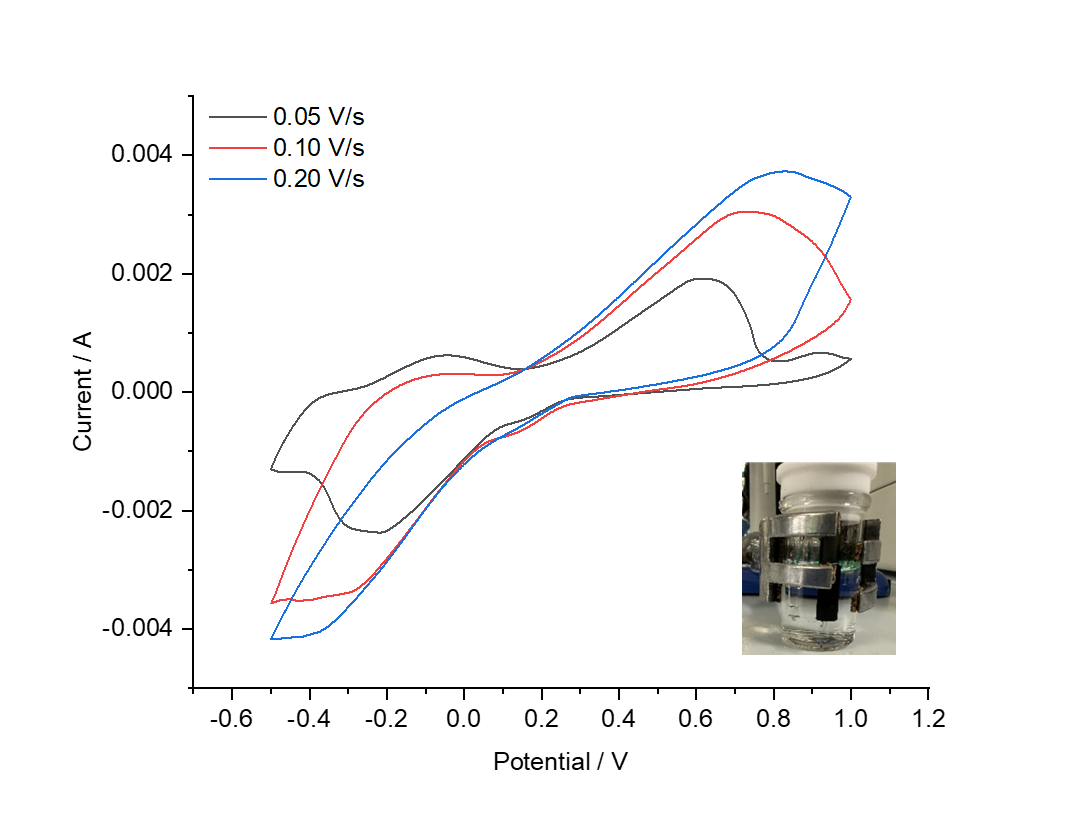


**Figure S7**. CV test of Ag/CuO drop casted on Au electrode.

The precursor ink was dropped on a gold electrode and dried about three weeks at room temperature to form Ag/CuO metallic mixture. The compound film has dark shiny metal surface. This electrode was tested at 3 scan rates in the same GLU NaOH solution.


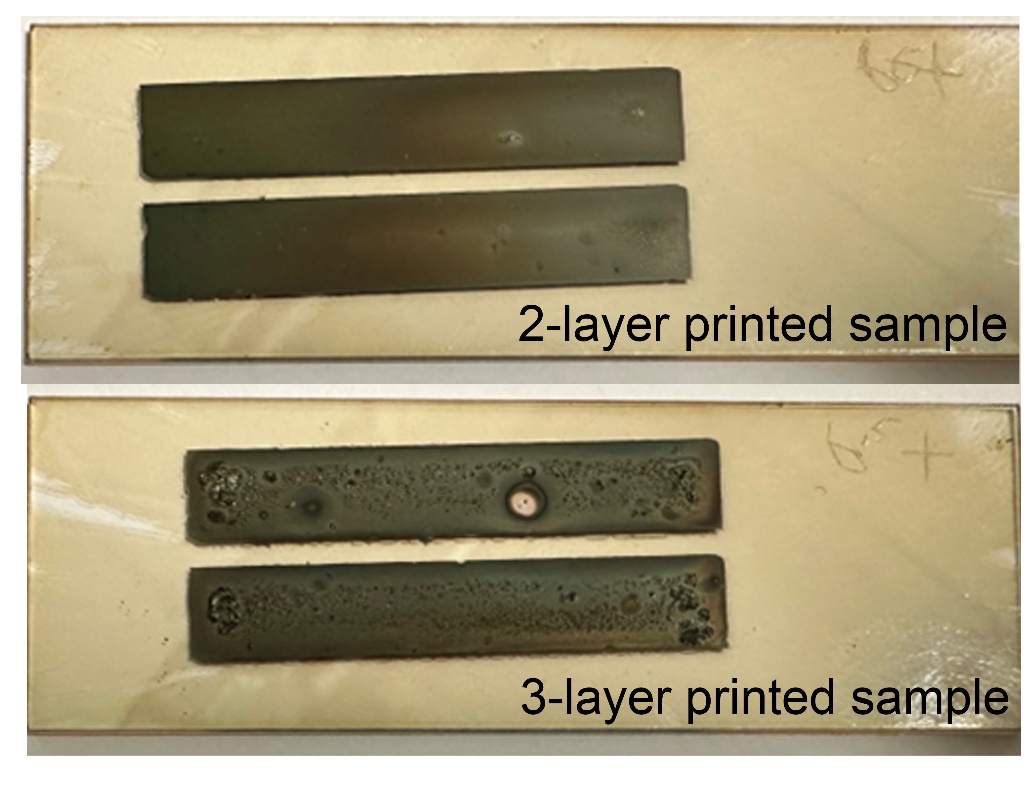


**Figure S8**. (a) Images of 2-layer printed sample (up) and 3-layer printed sample.


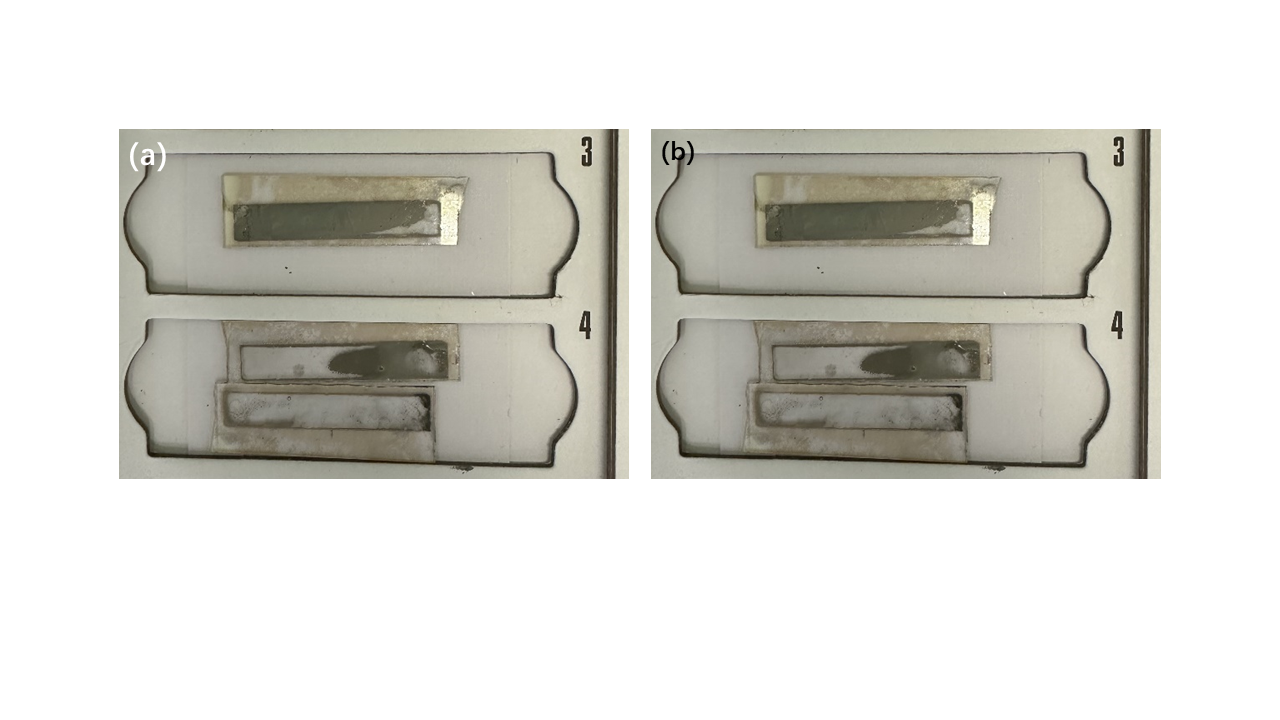


**Figure S9**. (a) Images of 2-layer printed sample (up) and 3-layer printed sample. (b) Images of 2-layer printed sample (up) and 3-layer printed sample after 2-hour CV test running.

As shown in Figure S9, after running for ~2 h, the film was still on the glass (2-layer printed sample) and still had electrochemical properties. In comparation with 2-layer printed sample, 3-layer printed sample was fragile and almost gone with CV running.

**SI 8. Inkjet printing (FUJI DIMATIX 2800)**


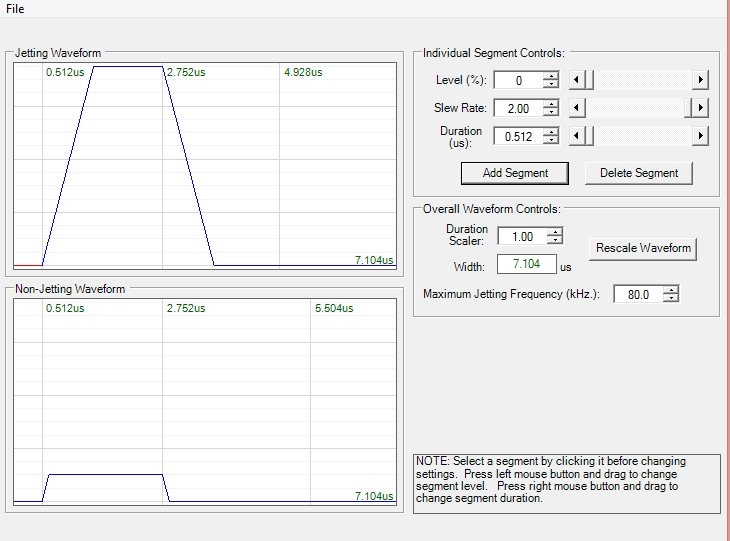


**Figure S10**. Jetting waveform setting for formulated hybrid ink in this study.


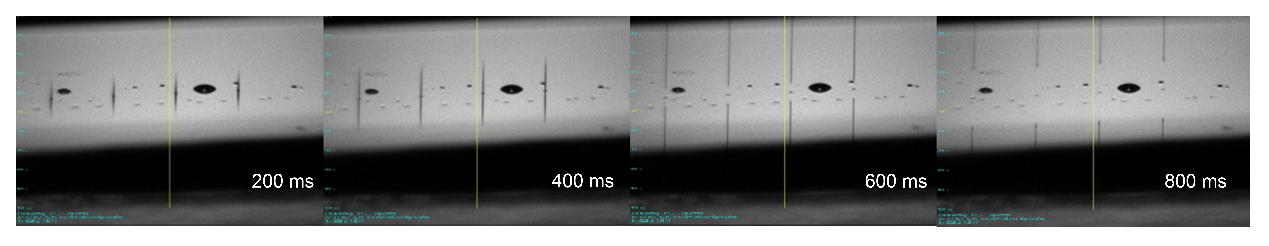


**Figure S11**. Picture of drop-watcher of formulated hybrid ink printed from nozzles.

**SI 9. SEM and EDS Characterization**

**SI 9.1 EDS analysis of cross-sections.**


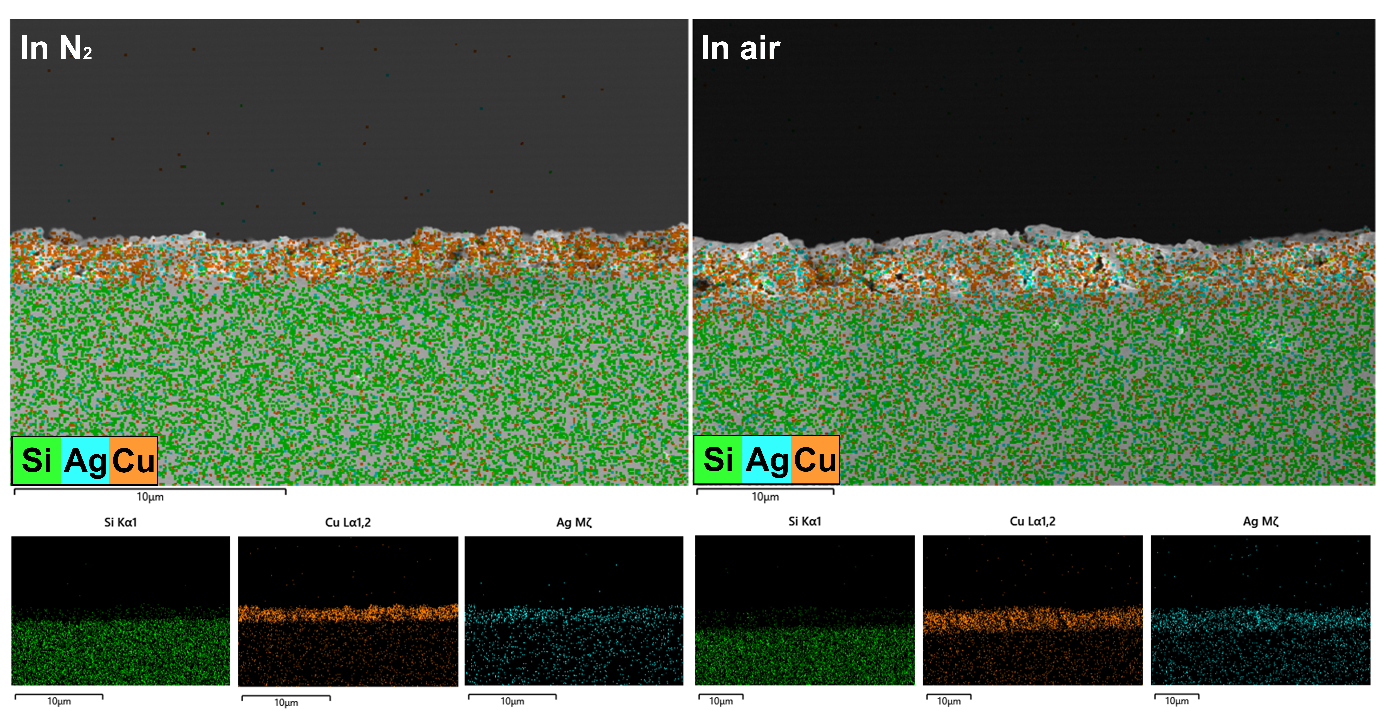


**Figure S12.** EDS mapping of cross-sections of 4-layer printed samples sintered in N_2_ and in air.

**SI 9.2 EDS of surface clusters of the sample sintered in air.**

| 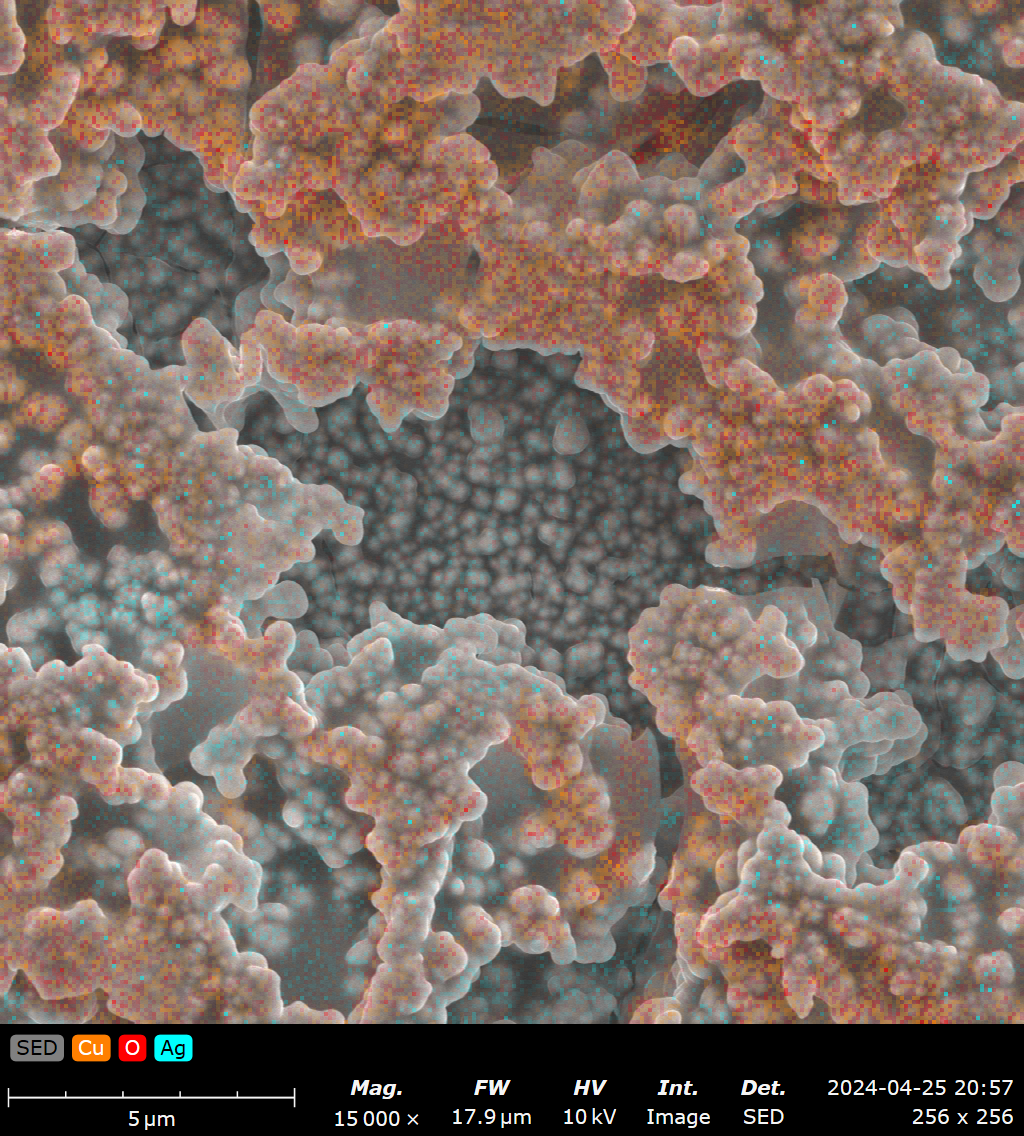 | \| **Element Number** \| **Element Symbol** \| **Element Name** \| **Atomic Conc.** \| **Weight Conc.** \| \| --- \| --- \| --- \| --- \| --- \| \| 29 \| Cu \| Copper \| 34.37 \| 35.08 \| \| 8 \| O \| Oxygen \| 33.06 \| 8.50 \| \| 47 \| Ag \| Silver \| 32.57 \| 56.43 \| \|  \|  \|  \|  \|  \| |
| --- | --- | --- | --- | --- | --- | --- | --- | --- | --- | --- | --- | --- | --- | --- | --- | --- | --- | --- | --- | --- | --- | --- | --- | --- | --- | --- |
|  |  |

FOV: 17.9 µm, Mode: 10kV - Image, Detector: SED, Time: APR 25 2024 20:57


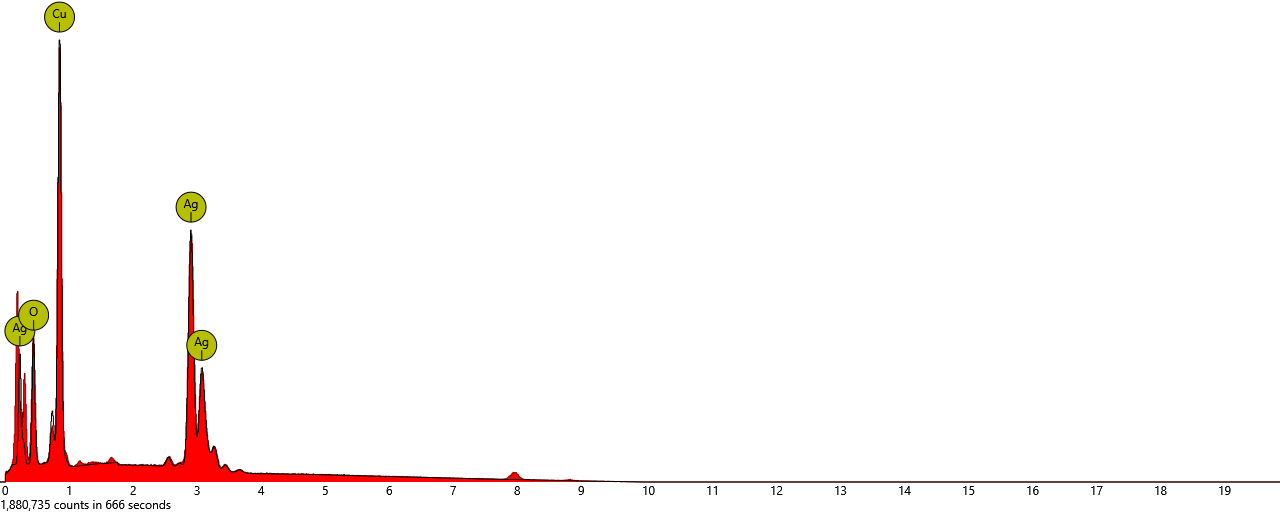


EDS of surface clusters of the sample sintered in air.

**SI 9.3 EDS of the layer under the surface clusters of the sample sintered in air.**

| 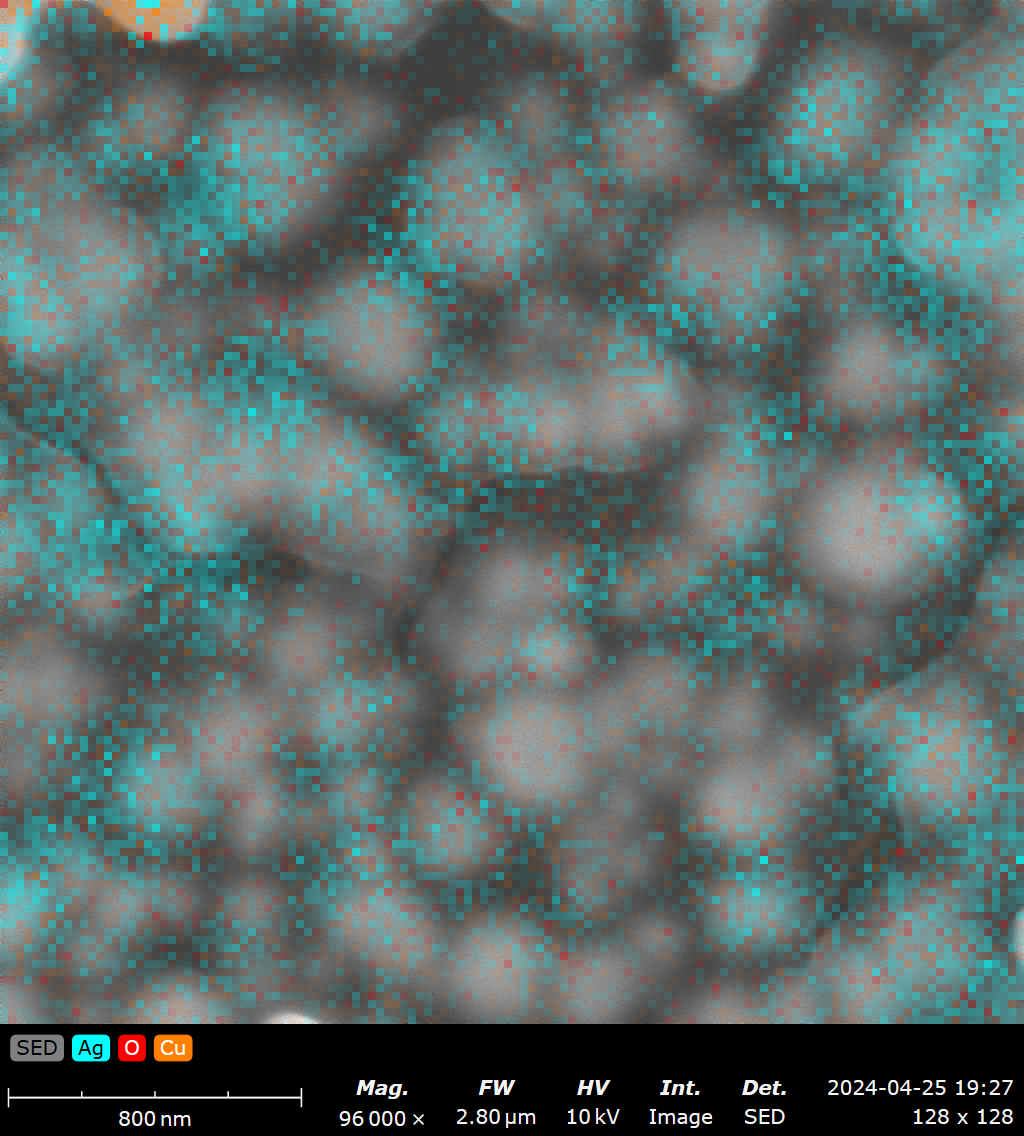 | \| **Element Number** \| **Element Symbol** \| **Element Name** \| **Atomic Conc.** \| **Weight Conc.** \| \| --- \| --- \| --- \| --- \| --- \| \| 47 \| Ag \| Silver \| 57.77 \| 80.63 \| \| 8 \| O \| Oxygen \| 24.96 \| 5.17 \| \| 29 \| Cu \| Copper \| 17.28 \| 14.21 \| \|  \|  \|  \|  \|  \| |
| --- | --- | --- | --- | --- | --- | --- | --- | --- | --- | --- | --- | --- | --- | --- | --- | --- | --- | --- | --- | --- | --- | --- | --- | --- | --- | --- |
|  |  |

FOV: 2.8 µm, Mode: 10kV - Image, Detector: SED, Time: APR 25 2024 19:27


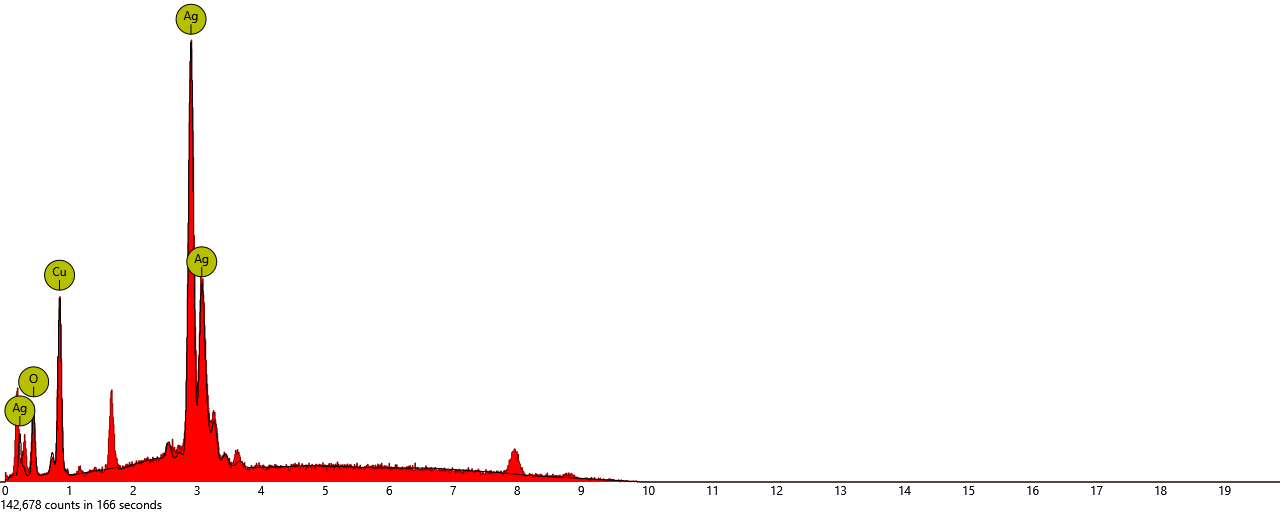


**SI 10. Visual view of the sintered patterns from top and bottom.**


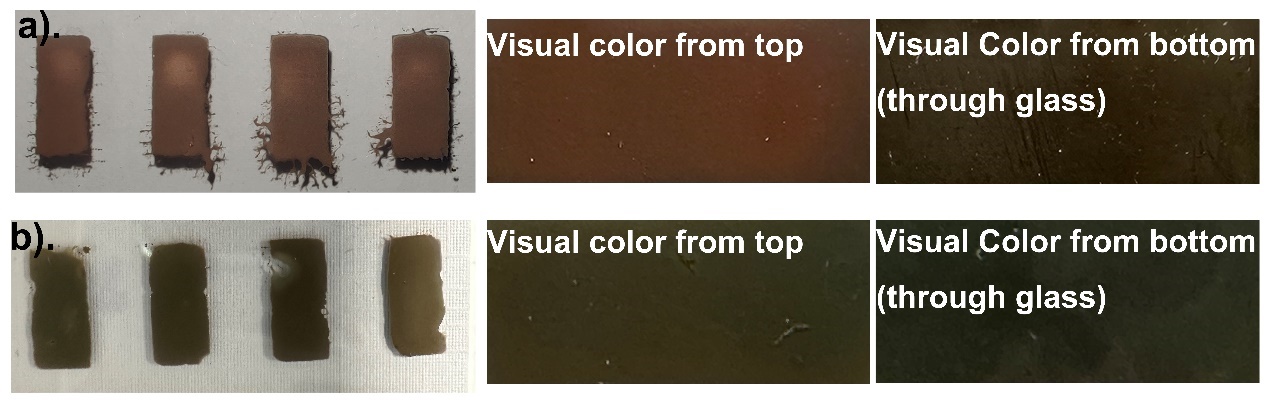


**Figure S13.** a) Visual view of the N_2_-sintered pattern from top and bottom. b) Visual view of the air-sintered pattern from top and bottom.

**SI 11. Thickness of air-sintered printed patterns**


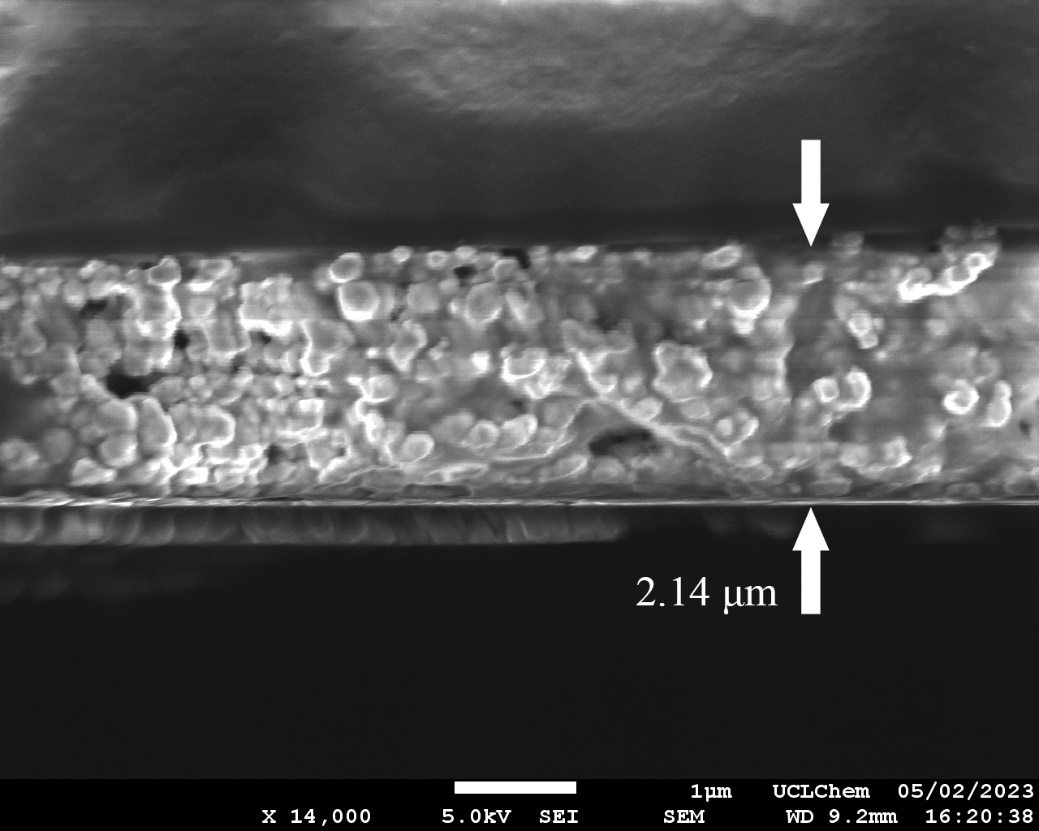


**Figure S14.** SEM image of cross-section of air-sintered single-layer printed pattern.


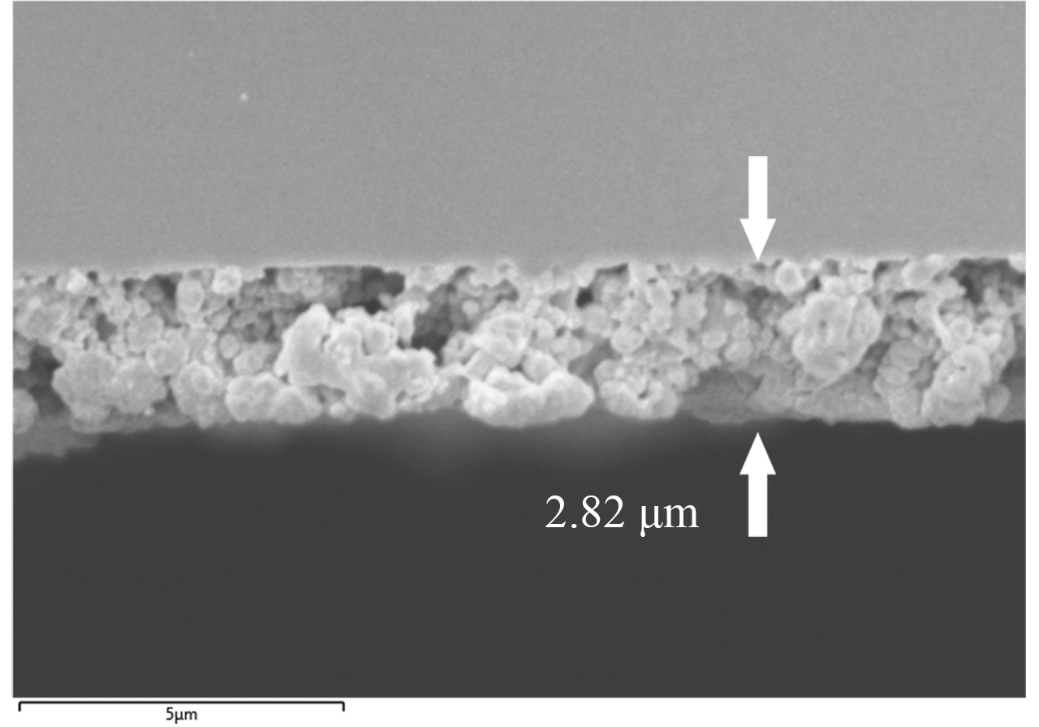


**Figure S15.** SEM image of cross-section of air-sintered 4-layer printed pattern.


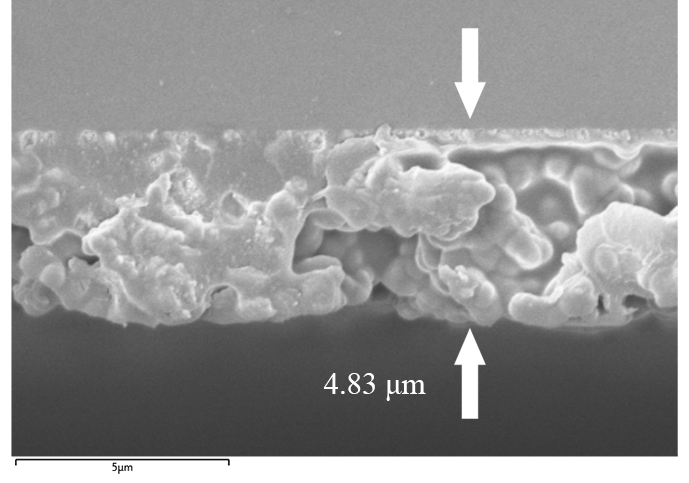


**Figure S16.** SEM image of cross-section of air-sintered 6-layer printed pattern.

**References**

1. M. J. Frisch, G. W. Trucks, H. B. Schlegel, G. E. Scuseria, M. A. Robb, J. R. Cheeseman, G. Scalmani, V. Barone, G. A. Petersson, H. Nakatsuji, X. Li, M. Caricato, A. V. Marenich, J. Bloino, B. G. Janesko, R. Gomperts, B. Mennucci, H. P. Hratchian, J. V. Ortiz, A. F. Izmaylov, J. L. Sonnenberg, Williams, F. Ding, F. Lipparini, F. Egidi, J. Goings, B. Peng, A. Petrone, T. Henderson, D. Ranasinghe, V. G. Zakrzewski, J. Gao, N. Rega, G. Zheng, W. Liang, M. Hada, M. Ehara, K. Toyota, R. Fukuda, J. Hasegawa, M. Ishida, T. Nakajima, Y. Honda, O. Kitao, H. Nakai, T. Vreven, K. Throssell, J. A. Montgomery Jr., J. E. Peralta, F. Ogliaro, M. J. Bearpark, J. J. Heyd, E. N. Brothers, K. N. Kudin, V. N. Staroverov, T. A. Keith, R. Kobayashi, J. Normand, K. Raghavachari, A. P. Rendell, J. C. Burant, S. S. Iyengar, J. Tomasi, M. Cossi, J. M. Millam, M. Klene, C. Adamo, R. Cammi, J. W. Ochterski, R. L. Martin, K. Morokuma, O. Farkas, J. B. Foresman and D. J. Fox, Gaussian 16 Rev. C.01 2016.
2. J. P. Perdew, K. Burke and M. Ernzerhof, Phys. Rev. Lett., 1997, 78, 1396–1396.
3. J. P. Perdew, K. Burke and M. Ernzerhof, Phys. Rev. Lett., 1996, 77, 3865–3868.
4. C. Adamo and V. Barone, J. Chem. Phys., 1999, 110, 6158–6170.
5. F. Weigend, Phys. Chem. Chem. Phys., 2006, 8, 1057–1065.
6. F. Weigend and R. Ahlrichs, Phys. Chem. Chem. Phys., 2005, 7, 3297–3305.
7. S. Grimme, S. Ehrlich and L. Goerigk, Journal of Computational Chemistry, 2011, 32, 1456–1465.
8. D. Andrae, U. Häußermann, M. Dolg, H. Stoll and H. Preuß, Theoret. Chim. Acta, 1990, 77, 123–141.
9. A. V. Marenich, C. J. Cramer and D. G. Truhlar, J. Phys. Chem. B, 2009, 113, 6378–6396.
